# Slingshot: cell lineage and pseudotime inference for single-cell transcriptomics

**DOI:** 10.1186/s12864-018-4772-0

**Published:** 2018-06-19

**Authors:** Kelly Street, Davide Risso, Russell B. Fletcher, Diya Das, John Ngai, Nir Yosef, Elizabeth Purdom, Sandrine Dudoit

**Affiliations:** 1 Division of Biostatistics, School of Public Health, University of California, Berkeley, CA USA; 2 Division of Biostatistics and Epidemiology, Department of Healthcare Policy and Research, Weill Cornell Medicine, 407 E 61st St, New York, 10065 NY USA; 3 Department of Molecular and Cell Biology, University of California, Berkeley, CA USA; 4 Department of Electrical Engineering and Computer Sciences, University of California, Berkeley, CA USA; 5 Department of Statistics, University of California, Berkeley, CA USA; 6 Helen Wills Neuroscience Institute, University of California, Berkeley, CA USA; 7 QB3 Berkeley Functional Genomics Laboratory, Berkeley, CA USA; 8 Center for Computational Biology, University of California, Berkeley, CA USA; 9 Berkeley Institute for Data Science, University of California, Berkeley, CA USA

**Keywords:** RNA-Seq, Single cell, Lineage inference, Pseudotime inference

## Abstract

**Background:**

Single-cell transcriptomics allows researchers to investigate complex communities of heterogeneous cells. It can be applied to stem cells and their descendants in order to chart the progression from multipotent progenitors to fully differentiated cells. While a variety of statistical and computational methods have been proposed for inferring cell lineages, the problem of accurately characterizing multiple branching lineages remains difficult to solve.

**Results:**

We introduce Slingshot, a novel method for inferring cell lineages and pseudotimes from single-cell gene expression data. In previously published datasets, Slingshot correctly identifies the biological signal for one to three branching trajectories. Additionally, our simulation study shows that Slingshot infers more accurate pseudotimes than other leading methods.

**Conclusions:**

Slingshot is a uniquely robust and flexible tool which combines the highly stable techniques necessary for noisy single-cell data with the ability to identify multiple trajectories. Accurate lineage inference is a critical step in the identification of dynamic temporal gene expression.

**Electronic supplementary material:**

The online version of this article (10.1186/s12864-018-4772-0) contains supplementary material, which is available to authorized users.

## Background

Traditional transcription assays, such as bulk microarrays and RNA sequencing (RNA-Seq), offer a bird’s-eye view of transcription. However, as they rely on RNA from a large number of cells as starting material, they are not ideal for examining heterogeneous populations of cells. Newly-developed single-cell assays can give us a much more detailed picture [1]. This higher resolution allows researchers to distinguish between closely-related populations of cells, potentially revealing functionally distinct groups with complex relationships [2].

For many systems, there are not clear distinctions between cellular states, but instead a smooth transition, where individual cells represent points along a continuum or *lineage*. Cells in these systems change states by undergoing gradual transcriptional changes, with progress being driven by an underlying temporal variable or *pseudotime*. For example, [3] examined the differentiation pattern of skeletal myoblasts, showing that their development into myocytes and mature myotubes follows a continuous lineage, rather than discrete steps. Inference of lineage structure has been referred to as *pseudotemporal reconstruction* and it can help us understand how cells change state and how cell fate decisions are made [3–5]. Furthermore, many systems contain multiple lineages that share a common initial state but branch and terminate at different states. These complex lineage structures require additional analysis to distinguish between cells that fall along different lineages [6–10].

Several methods have been proposed for the task of pseudotemporal reconstruction, each with their own set of strengths and assumptions. We describe a few popular approaches here; for a thorough review see [11, 12]. One of the most well-known methods is Monocle [3], which constructs a minimum spanning tree (MST) on cells in a reduced-dimensionality space created by independent component analysis (ICA) and orders cells via a PQ tree along the longest path through this tree. The direction of this path and the number of branching events are left to the user, who may examine a known set of marker genes or use time of sample collection as indications of initial and terminal cell states. The more recent Monocle 2 [8] uses a different approach, with dimensionality reduction and ordering performed by reverse graph embedding (RGE), allowing it to detect branching events in an unsupervised manner. The methods Waterfall [10] and TSCAN [7] instead determine the lineage structure by clustering cells in a low-dimensional space and drawing an MST on the cluster centers. Lineages are represented by piecewise linear paths through the tree, providing an intuitive, unsupervised method for identifying branching events. Pseudotimes are calculated by orthogonal projection onto these paths, with the identification of the direction and of the cluster of origin again left to the user. Other approaches use smooth curves to represent development, but are naturally limited to non-branching lineages. For example, Embeddr [5] uses the principal curves method of [13] to infer lineages in a low-dimensional space obtained by a Laplacian eigenmap [14]. Yet another class of methods uses robust cell-to-cell distances and a pre-specified starting cell to determine pseudotime. For instance, diffusion pseudotime (DPT) [6] uses a weighted *k* nearest neighbors (*k*NN) graph on cells and calculates distances using transition probabilities over random walks of arbitrary length. Similarly, Wishbone [9], an extension of Wanderlust [4], uses an ensemble of *k*NN graphs on cells along with a randomly selected group of waypoints to iteratively refine stable distance estimates. Finally, other methods take a model-based approach to detecting branching events. GPfates [15] uses a Gaussian process latent variable model (GPLVM) and overlapping mixtures of Gaussian processes (OMGP) to infer trajectories and pseudotimes. A similar method, DeLorean [16], uses a single GPLVM to infer pseudotimes along a single trajectory. And the mixtures of factor analysers (MFA) method [17] takes a hierarchical Bayesian approach, using Markov chain Monte-Carlo (MCMC) to sample from the posterior of a fully generative model that includes branch identities. See Table 1 for a summary of existing methods.

**Table 1:** Summary of existing lineage and pseudotime inference methods

	Dimensionality reduction	Cluster based	Graph	Pseudotime calculation	Branching	Supervision
Diffusion Pseudotime	Diffusion maps	No	Weighted k-NN graph on cells	Transition probabilities over arbitrary length random walks	Yes	Starting cell
Embeddr	Laplacian eigenmaps	No	N/A	Principal curve, orthogonal projection	No	Path direction^1^, subsetting^2^
Monocle	ICA	No	MST on cells	Diameter path, PQ trees	Yes^3^	Path direction^1^, number of lineages
Monocle 2	Reversed graph embedding	No	Principal graph on cells	Distance to root	Yes	Starting cluster
TSCAN	PCA	Yes	MST on clusters	Cluster centers, orthogonal projection	Yes	Starting cluster
Waterfall	PCA	Yes	MST on clusters	Cluster centers, orthogonal projection	Yes^4^	Path direction^1^
Wishbone	Diffusion maps	No	Ensemble of k-NN graphs on cells	Distance refinement by waypoints	Yes^5^	Starting cell
Slingshot	Any	Yes	MST on clusters	Simultaneous principal curves, orthogonal projection	Yes	Starting cluster, end clusters (optional)

^1^Some methods infer a single path or backbone and rely on the user to assign its directionality

^2^Methods that do not detect branching events require manually subsetting the data down to a single lineage

^3^Monocle does not detect the number of branching events, the number of lineages must be supplied by the user

^4^Waterfall detects branching events, but requires subsetting to a single lineage for pseudotime calculation

^5^Wishbone can only detect a single branching event (two lineages)

**Fig. 1:**
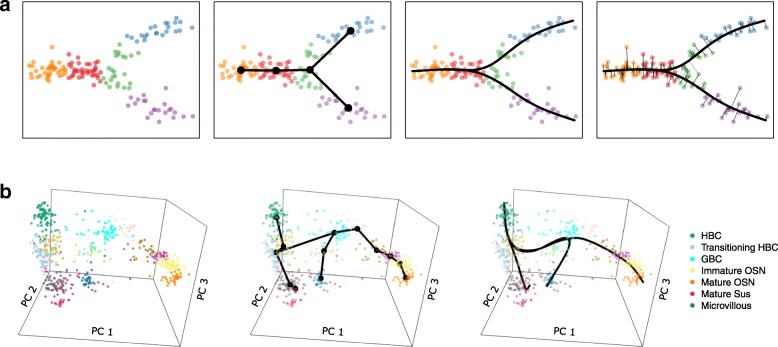
Schematics of Slingshot’s main steps. The main steps for Slingshot are shown for: Panel (**a**) a simple simulated two-lineage two-dimensional dataset and Panel (**b**) the single-cell RNA-Seq olfactory epithelium three-lineage dataset of [26] (see Results and discussion for details on dataset and its analysis). Step 0: Slingshot starts from clustered data in a low-dimensional space (cluster labels indicated by color). For Panel (**b**), the plot shows the top three principal components, but Slingshot was run on the top five. Step 1: A minimum spanning tree is constructed on the clusters to determine the number and rough shape of lineages. For Panel (**b**), we impose some constraints on the MST based on known biology. Step 2: Simultaneous principal curves are used to obtain smooth representations of each lineage. Step 3: Pseudotime values are obtained by orthogonal projection onto the curves (only shown for Panel (**a**))

Here, we introduce Slingshot, a novel lineage inference tool designed for multiple branching lineages. Slingshot combines highly stable techniques necessary for noisy single-cell data with the flexibility to identify multiple lineages with varying levels of supervision. Slingshot consists of two main stages: 1) the inference of the global lineage structure and 2) the inference of pseudotime variables for cells along each lineage (Fig. 1). Like other methods [7, 10], Slingshot’s first stage uses a cluster-based MST to stably identify the key elements of the global lineage structure, i.e., the number of lineages and where they branch (Fig. 1, **Step 1**). This allows us to identify novel lineages while also accommodating the use of domain-specific knowledge to supervise parts of the tree (e.g., terminal cellular states). For the second stage, we propose a novel method called *simultaneous principal curves*, to fit smooth branching curves to these lineages, thereby translating the knowledge of global lineage structure into stable estimates of the underlying cell-level pseudotime variable for each lineage (Fig. 1, **Step 2**). The Slingshot method is implemented in the open-source R package slingshot (available from the GitHub repository https://github.com/kstreet13/slingshot) to be released through the Bioconductor Project (http://www.bioconductor.org).

In addition to Slingshot’s core methodological components described above for lineage and pseudotime inference, we note the importance of upstream analysis choices. Indeed, most pseudotemporal reconstruction methods will either explicitly or implicitly require certain choices at previous steps in the workflow. Dimensionality reduction, for example, helps in reducing the amount of noise in the data and in visualization, but a variety of approaches are available, with a potentially large impact on the final result (Additional file 1: Figure S7). Monocle recommends ICA or DDRTree, Waterfall and TSCAN use principal component analysis (PCA), Embedder uses Laplacian eigenmaps [14], and Wishbone uses diffusion maps for analysis and t-distributed stochastic neighbor embedding (t-SNE) [18] for visualization (Table 1). Given the great diversity of data being generated by single-cell assays, it seems unlikely that there is a one-size-fits-all solution to the dimensionality reduction problem; likewise for normalization and clustering. These analysis steps are very important and because different lineage inference methods make different upstream choices, they can be difficult to compare. Slingshot does not specify these upstream methods, but is instead designed with flexibility and modularity in mind, to easily integrate with the normalization, dimensionality reduction, and clustering methods deemed most appropriate for a particular dataset. Our recommended single-cell RNA-Seq data analysis workflow, implemented in Bioconductor R packages, is described in [19]: the pipeline includes the data-adaptive selection of a normalization procedure (scone package; [20]), dimensionality reduction using a zero-inflated negative binomial model (zinbwave package; [21]), and resampling-based sequential ensemble clustering (RSEC; clusterExperiment package; [22]).

## Results and discussion

Slingshot divides the problem of multiple lineage inference into two stages:

- **1.**  Identification of *lineages*, i.e., ordered sets of cell clusters, where all lineages share a starting cluster and each leads to a unique terminal cluster. This is achieved by constructing an MST on clusters of cells.
- **2.**  For each lineage, identification of *pseudotimes*, i.e., a one-dimensional variable representing each cell’s transcriptional progression toward the terminal state. This is achieved by a method which extends principal curves [13] to the case of multiple branching lineages.

One of the main challenges of single-cell RNA-Seq data analysis is the high level of variability. In addition to the host of biological and technical sources of variation that can affect any (bulk) RNA-Seq experiment, single-cell data may contain effects from transcriptional bursting [23, 24] and drop-out [25]. We therefore believe that robustness to noise, unwanted technical effects, and preprocessing are important characteristics of a lineage inference method. Slingshot provides the flexibility to capture complex lineage structures along with the stability needed for working with noisy single-cell data.

### Real datasets

**Fig. 2:**
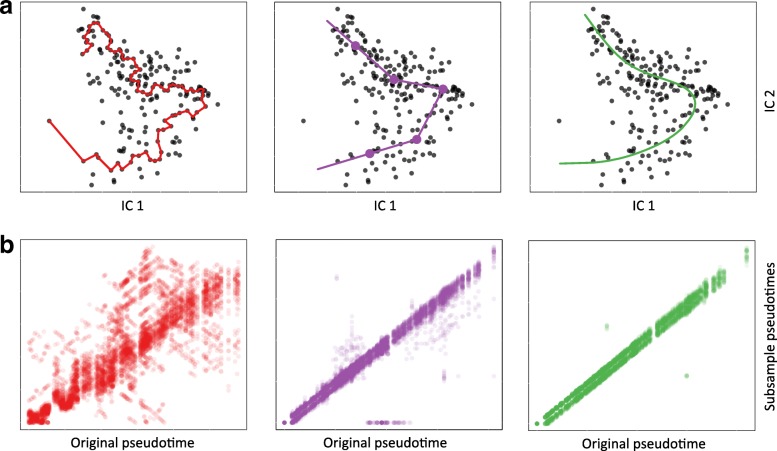
Robustness of lineage and pseudotime inference methods: HSMM dataset. We examine the stability of three lineage and pseudotime inference approaches on the single-lineage HSMM dataset of [3], showing how each method orders the cells for the original dataset, as well as for 50 subsamples of the data. Panel (**a**): Monocle identifies the longest path through an MST constructed on all cells (red). Waterfall and TSCAN cluster cells and connect cluster centers with an MST (purple, clustering performed by *k*-means with *k*=5). Embeddr and Slingshot order cells using a principal curve, i.e., a non-linear fit through the data (green). As in [3], dimensionality reduction is performed by ICA. Panel (**b**): Scatterplots of pseudotimes based on 50 subsamples of the data vs. pseudotimes for the original dataset. Subsamples were generated in a bootstrap-like manner, by randomly sampling *n* times, with replacement from the original cell-level data and retaining only one instance of each cell. Thus, subsamples were of variable sizes, but contained on average about 63% of the original cells. The cluster-based MST method occasionally detected spurious branching events and, for the purpose of visualization, cells not placed along the main lineage were assigned a pseudotime value of 0

**Robustness to noise.** We first examined the stability of a few well-known methods using a subset of the human skeletal muscle myoblasts (HSMM) dataset of [3] comprising a single lineage. In Fig. 2, we illustrate each method’s ordering of the full set of 212 cells and show how consistently it orders cells over 50 subsamples (bootstrap samples with duplicates removed). The Monocle procedure, which constructs an MST on individual cells and orders them according to a PQ tree along the longest path of the MST, was the least stable of the methods we compared. The path drawn by Monocle was highly variable and sensitive to even small amounts of noise; this instability has been previously discussed in [7]. In contrast, other methods which emphasize stability in the construction of their primary trajectory and obtain pseudotime values based on orthogonal projection produced much more stable orderings.

Both the cluster-based MST method [7, 10] and the principal curve method [5, 13] demonstrated stability over the bootstrap-like samples shown in Fig. 2b. However, due to the vertices of the piecewise linear path drawn by the cluster-based MST, multiple cells will often be assigned identical pseudotimes, corresponding to the value at the vertex. The principal curve approach was the most stable method, but on more complex datasets, it has the obvious limitation of only characterizing a single lineage. It is for this reason that we chose to extend principal curves to accommodate multiple branching lineages.

**Multiple lineage inference.** One of the biggest challenges in lineage inference is determining the number and location of branching events. Some methods introduce simplifying assumptions or restrictions on discovery; for example, requiring the user to pre-specify the number of lineages or limiting the model space to only one or two. Slingshot allows for multiple lineage detection without pre-specifying or limiting the number of lineages. Instead, Slingshot provides a framework for optional incorporation of localized prior biological knowledge that does not restrict other parts of the tree or introduce global specifications. As with the specification of an initial cluster, users may specify a certain number of terminal clusters, which will be restricted to a single edge in the cluster-based MST.

**Fig. 3:**
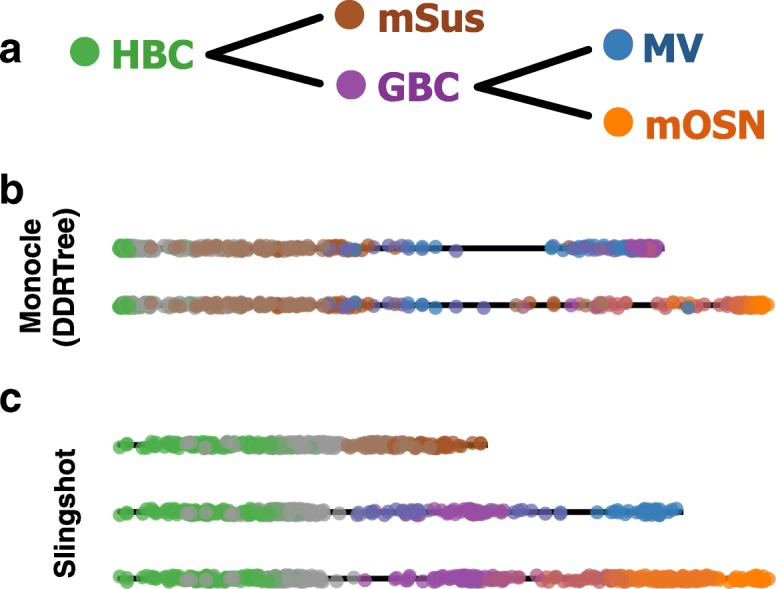
Multiple lineage inference: OE dataset. Pseudotime variables for each lineage inferred by Slingshot and Monocle 2 on the three-lineage OE dataset of [26]. Panel (**a**): Known biological relationships between cell types. Panel (**b**): For Monocle 2, we used the DDRTree algorithm to obtain a two-dimensional (or five-dimensional, see Additional file 1: Figure S3d) representation of the data and selected the starting state based on the highest percentage of cells from the HBC cluster. Panel (**c**): For Slingshot, we used the top five PCs and clustered cells by RSEC, as in the original article. The HBC cluster was specified as the origin and the mSus cluster as an endpoint; other endpoints were identified without supervision

This local supervision was used in the analysis of the olfactory epithelium (OE) data of [26] to mark mature sustentacular (mSus) cells, microvillous (MV) cells, and mature olfactory sensory neurons (mOSN) as terminal states, though only the first had an effect on the eventual cluster-based MST. Slingshot’s resulting lineage structure established the order of the two bifurcations, which was later validated. Specifically, it was demonstrated that sustentacular cells are produced via direct conversion of horizontal basal cells (HBC), whereas microvillous and neuronal cells require an intermediate, proliferative state (see Fig. 3a for a summary of validated relationships between cell types).

We also note that the OE lineage structure could not have been recovered using standard Euclidean distances between cluster centers (Additional file 1: Figure S1c), as in Waterfall and TSCAN. By failing to utilize the shapes of the clusters, the standard Euclidean distance identified a spurious branching event very early on in HBC differentiation. Slingshot allows the use of a shape-sensitive distance measure inspired by the Mahalanobis distance [27], which scales the distance between cluster centers based on the covariance structure of the two clusters.

While Slingshot identified lineages consistent with prior biological knowledge, other lineage detection methods did not. Monocle 2 only identified two lineages, one of which terminates in globose basal cells (GBC), a known transition state, and both of which contain sustentacular cells and microvillous cells, known endpoints of separate lineages (Fig. 3). TSCAN also produced only two lineages with similar issues (Additional file 1: Figure S3f). Given the proper number of lineages, Monocle also misidentified GBCs as a terminal state, but correctly identified lineages terminating in mOSNs and mSus cells (Additional file 1: Figure S3e). Diffusion pseudotime identified these endpoints as well, but it additionally found several spurious lineages (nine in total, Additional file 1: Figure S3j). Finally, Wishbone is limited in implementation to only two lineages, but even when we restricted the analysis to only the sustentacular and neuronal lineages (as identified by Slingshot), it still failed to accurately characterize this single branching event (Additional file 1: Figure S4c).

In Additional file 1: Figure S2, we show that, in addition to capturing complex multi-lineage structures, Slingshot is also able to correctly detect a single lineage and two bifurcating lineages, respectively, in the datasets of [3, 10]. In both cases, Slingshot’s final pseudotime variables are highly correlated with those found in the original publications, but do not rely on user specification of the number of lineages nor on subsetting the data, as in the case of Waterfall.

### Simulation study

**Fig. 4:**
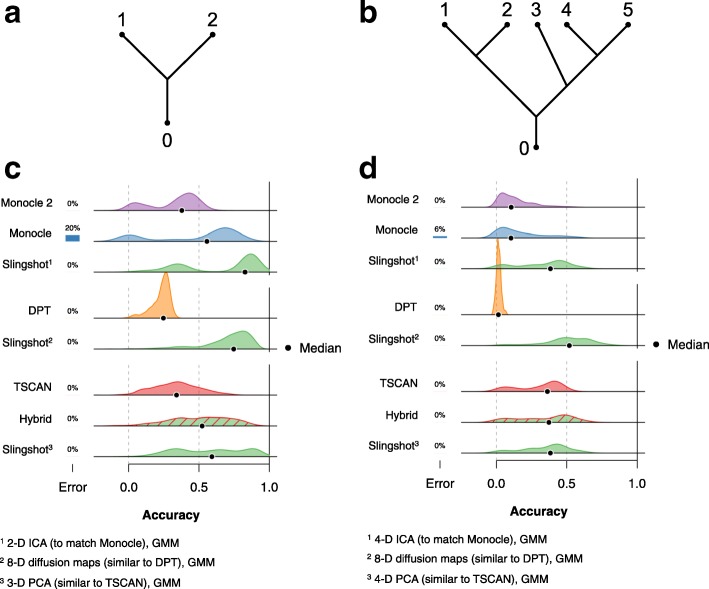
Comparison of accuracy scores for lineage and pseudotime inference methods: Simulated datasets. Gaussian kernel density plots of accuracy scores show how five lineage inference methods performed on a series of simulated datasets with two different topologies: Panels (**a**,**c**) two lineages and Panels (**b**,**d**) five lineages. In both settings, the simulated data contained variable numbers of cells and levels of noise. Bars to the left of each density plot represent the percentage of datasets on which a method returned an error. Errors are treated as 0 values for calculating the median score, but are not included in the density estimates. Monocle, Monocle 2, DPT, and TSCAN were implemented in several ways and these densities represent the best results obtained by each method. Slingshot was implemented with various dimensionality reduction techniques, chosen to match the best-case settings of the other methods and with clusters assigned by Gaussian mixture modeling (GMM). See Simulation study for the definition of accuracy scores based on Kendall’s rank correlation coefficient and Additional file 1 for details on simulation scenarios

**Design.** In order to make a more quantitative comparison of different lineage inference methods and examine Slingshot’s robustness to upstream computational choices, we conducted a simulation study with synthetic datasets generated using the Bioconductor R package splatter [28]. In the first part of the study, all simulated datasets sconsisted of an initial path that bifurcates into two distinct lineages (Fig. 4a). In the second part of the study, each dataset was simulated from a more complex branching structure, with five distinct lineages (Fig. 4b). For the two-lineage portion of the simulation study, 1200 synthetic datasets were generated and for the five-lineage portion, 300 datasets were simulated. The number of cells in the datasets and the signal-to-noise ratio were varied; parameters defining the marginal distributions of the expression measures for both genes and samples were learned from the dataset of [3] (see Additional file 1: Figure S6 for parameter values used by splatter). Transcript-level counts were obtained from the conquer repository [29] and aggregated into gene-level counts. Unless otherwise noted, datasets were full-quantile-normalized prior to lineage inference. See “Methods” section for complete details.

The accuracy of inferred pseudotimes was measured as follows: for each true lineage, identify the best match amongst all inferred lineages, according to the Kendall rank correlation coefficient between the true and inferred pseudotime variables. Averaging these values over all true lineages yields the accuracy score for a particular method on a particular dataset. As with the standard Pearson correlation coefficient, the Kendall rank correlation coefficient achieves values between -1 and 1, with values closer to one indicating better agreement between the inferred and true pseudotimes.

Slingshot was applied with various upstream dimensionality reduction and clustering techniques, allowing us to assess their impact on the accuracy of the resulting pseudotime variables. Existing lineage inference methods were also applied to each dataset in order to compare their performance. For each of these existing methods, multiple strategies were implemented and the best-performing strategy was selected as the representative of that method. We then compared these best-case runs to a similar implementation of Slingshot, matched for dimensionality reduction and clustering, when possible.

Monocle and Monocle 2 were implemented using a range of values for *J*^′^, the size of the reduced-dimensional space, and two techniques for selecting genes. The first, suggested in the Monocle vignette, used genes with the 100 highest loadings along the top *J*^′^ principal components. The second selected genes with either the 5000 highest means or variances of log-counts; this is comparable to using all the genes, but less computationally burdensome, as Monocle and Monocle 2 tended to be the slowest of the methods we examined (other methods used the full set of genes). Since it cannot detect branching, Monocle was always given the correct number of lineages. TSCAN was implemented both with and without the recommended preprocessing step and with this step in place of full-quantile normalization. Additionally, we present results from a hybrid method which uses TSCAN for dimensionality reduction and clustering before using Slingshot for pseudotime inference, in order to study the combined impact of replacing Euclidean distances by covariance-weighted distances for the cluster-based MST and piecewise linear paths by simultaneous principal curves. Diffusion pseudotime (DPT) was implemented with default parameter values, but multiple strategies had to be implemented in order to account for variable amounts of missing information in the results. The “DPT-Full” strategy uses every branching event reported, often resulting in cells being dropped due to missing branch information. Other strategies, such as “DPT-2”, “DPT-3”, etc., use only the highest-level branching events, producing the specified number of lineages, with “DPT-1” ignoring branching events altogether. For additional details on how these methods were implemented, see the Simulation Study Design and Results section in Additional file 1 of the Supplementary Information. Finally, we note that the bifurcation events present in datasets generated by splatter are “sharp” rather than curved (see Additional file 1: Figure S7), which may disadvantage methods that assume smoothness, such as Slingshot and DPT.

**Comparison of methods.** In the two-lineage case, most of the Monocle strategies performed well, often producing a bimodal distribution of accuracy scores with one peak around 0 and a larger peak at or above 0.5 (Additional file 1: Figure S10). However, Monocle also returned an error more often than any other method and these errors seem to be associated with larger sample sizes (Additional file 1: Figure S11). We also note that Monocle was always provided the correct number of lineages, which most other methods were not. Among the strategies we implemented, the highest median accuracy score was achieved with the larger gene set (selected by the highest 5000 means and variances), with two-dimensional ICA. We therefore compared these results to Slingshot accuracy scores using two-dimensional ICA and clustering by Gaussian mixture modeling (GMM) in Fig. 4b. Slingshot’s distribution of accuracy scores was similarly bimodal, but with both peaks shifted slightly higher.

Compared to Monocle, Monocle 2 was more consistent, but less accurate overall. It rarely returned scores close to 0 and showed considerably less bimodality, especially with four- or five-dimensional RGE (Additional file 1: Figure S10). The lower overall accuracy scores may be due, in part, to the large number of spurious branching events it identified; in the synthetic datasets with two lineages, Monocle 2 identified four or more lineages 80.3*%* of the time. Unlike other methods, increasing the number of cells in the dataset did not improve the performance of Monocle 2, but actually resulted in even more spurious lineages being found. For datasets of more than 360 cells, Monocle 2 failed to find the correct number of lineages in any simulation, sometimes finding as many as 16 (Additional file 1: Figure S14). As the highest median accuracy score for Monocle 2 was also produced with the larger gene set and *J*^′^=2, we compare it to the same set of Slingshot results in Fig. 4b.

As discussed previously, Diffusion Pseudotime suffered from a considerable proportion of cells with missing branch assignments, leading to artificially low accuracy scores. In both the two- and five-lineage cases, the highest median accuracy score was achieved by the DPT-1 strategy, which did not make use of the branching information. We examined this issue in the two-lineage case, looking at the highest-level branching event, which should theoretically divide the cells into three groups: one prior to the branching event and two after it. On average, 44.1*%* of cells were not assigned a group. There was no noticeable relationship between this percentage and sample size, but the percentage of unassigned cells did decrease modestly with increased signal in the data. We compared DPT results to Slingshot results with eight-dimensional diffusion maps (the highest dimensionality we implemented) and Gaussian mixture modeling in both Figs. 4b and d.

TSCAN with full-quantile normalization produced accuracy scores comparable to Monocle 2. When run with the recommended preprocessing step, TSCAN did slightly worse, particularly in the absence of full-quantile normalization. The highest median accuracy score was produced by the hybrid method with Slingshot, which used full-quantile normalization with no additional preprocessing. For a more complete comparison, Fig. 4b also shows the best “pure” TSCAN strategy and Slingshot results with three-dimensional PCA and GMM clustering. We also note that our comparison may be slightly unfavorable for TSCAN, because it has built-in methods for selecting both *J*^′^ (the reduced number of dimensions) and *K* (the number of clusters). This user-friendliness unfortunately means that there are fewer parameters over which we could try different strategies, hence our implementations without full-quantile normalization and as part of a hybrid method with Slingshot. For complete results of all strategies implemented on the two-lineage datasets, see Additional file 1: Figure S10.

In the second part of the simulation study, the more complex five-lineage structure led to lower scores for most methods, with the notable exception of TSCAN, which produced a marginally higher median accuracy score than in the two-lineage case (Fig. 4d). The methods which do not make use of a cluster-based MST had the poorest performance in this setting, while TSCAN and Slingshot fared slightly better. We compared the TSCAN results to those of the hybrid method and Slingshot with 4-dimensional PCA and GMM. Again, the best strategies for Monocle and Monocle 2 made use of the larger gene sets, this time with 4-dimensional ICA and 5-dimensional RGE, respectively. We compared this to Slingshot results using 4-dimensional ICA and GMM. Monocle 2 continued to identify a large number of spurious lineages and there was still strong correlation between sample size and the number of lineages it inferred. For complete results of all strategies implemented on the five-lineage datasets, see Additional file 1: Figure S12.

Unlike the two-lineage case, the five-lineage topology is asymmetric, meaning that some lineages were harder to characterize than others. Nonetheless, Slingshot and TSCAN still generally outperformed other methods across all lineage types; for a full breakdown of all methods’ accuracy scores on all lineages, see Additional file 1: Figure S15.

**Robustness to clustering.** Although Slingshot uses cluster labels for the identification of lineages and branching events, the subsequent use of simultaneous principal curves to obtain pseudotimes makes its final results quite robust to the choice of clustering method. In comparison, methods that project cells directly onto a cluster-based MST, such as TSCAN and Waterfall, are more dependent on the initial clustering and, particularly, on the locations of the cluster centers, which can be highly variable (Additional file 1: Figure S17).

**Fig. 5:**
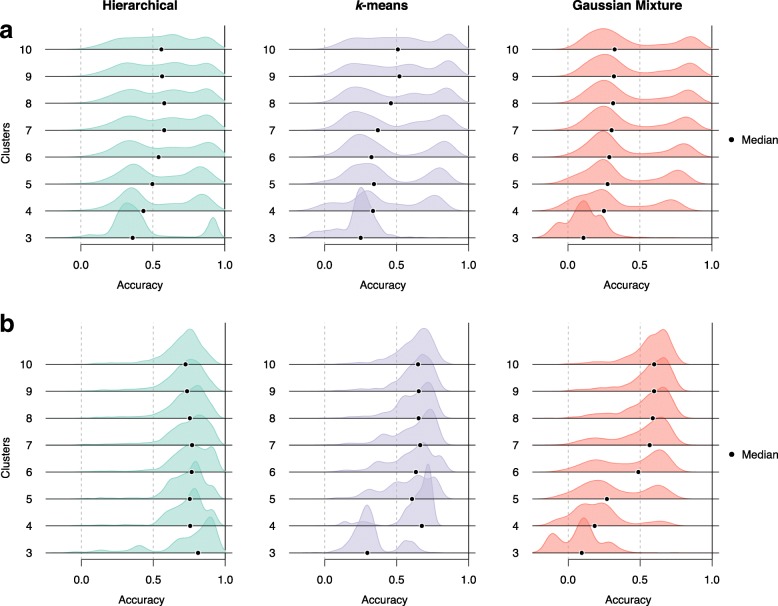
*Robustness of Slingshot pseudotimes to clustering method: Simulated two-lineage datasets.* Gaussian kernel density plots of accuracy scores for different clustering methods (columns) and numbers of clusters (rows) based on simulated data with two lineages. Clustering was performed using hierarchical clustering, *k*-means, and Gaussian mixture modeling, with a range of values for the number of clusters, *K*. Principal component analysis was used for dimensionality reduction with two values for the number of components *J*^′^: in Panel (**a**), three-dimensional PCA produced highly variable scores, while in Panel (**b**), four-dimensional PCA produced consistently high scores. Both panels show that Slingshot produces similar distributions of accuracy scores over a range of values for *K*. However, when *K*=3, Slingshot is often unable to detect the branching event and the resulting pseudotimes imperfectly match either true lineage. With more clusters, we see consistently accurate results. At higher values of *K* (not shown), accuracy scores begin to degrade slowly, as Slingshot begins to overfit and identify more spurious branching events. See “Simulation study” section for the definition of accuracy scores based on Kendall’s rank correlation coefficient and Additional file 1 for details on simulation scenarios

We examined Slingshot’s robustness to the choice of clustering method using the simulated datasets of the two-lineage topology and found that the particular clustering method is generally less important than the choice of *K*, the number of clusters (Fig. 5). For the three methods examined (hierarchical clustering, *k*-means, and Gaussian mixture modeling), Slingshot consistently produced similar distributions of accuracy scores over a range of values for *K* (Fig. 5). This stability held whether we used a “good” dimensionality reduction, which generally led to high accuracy scores (4-D PCA), or not (3-D PCA). For a similar examination of the impact of different dimensionality reduction techniques, see Additional file 1: Figure S8.

This robustness is a result of Slingshot’s use of simultaneous principal curves to smooth the cluster-based MST, but there is still an important relationship between clusters and lineage inference. In extreme cases with too few clusters, the cluster-based MST may fail to identify a branching event, and with too many, it may identify spurious branching events. These issues are common to all cluster-based MST methods and not mitigated by the use of simultaneous principal curves. However, when the correct global lineage structure can be approximately identified, simultaneous principal curves allow for increased stability and decreased reliance on particular clustering results.

## Conclusions

We have introduced a novel method, Slingshot, for lineage and pseudotime inference in single-cell genomics data. Because Slingshot breaks the inference problem into two steps, we are able to make use of appropriate methods for each task and avoid the common trade-off between stability and the flexibility to detect complex structures. Using a cluster-based MST for lineage inference allows Slingshot to identify potentially complex global patterns in the data without being overly sensitive to individual data points. And our novel simultaneous principal curves method for pseudotime inference extends the stability and robustness properties of principal curves to the case of multiple branching lineages.

We demonstrated Slingshot’s ability to correctly detect a single lineage and two branching lineages, using the data of [3, 10] respectively (Additional file 1: Figure S2), and in both cases it produced results similar to those found and validated in the original publications. Furthermore, using the olfactory epithelium dataset of [26], we demonstrated that with minimal supervision, Slingshot could correctly identify a complex three-lineage structure that other methods could not. Our simulation study showed that this advantage persisted over a range of input data types. For varying levels of noise and numbers of cells, Slingshot was able to infer accurate pseudotime variables more consistently than any other method, even with the same upstream dimensionality reduction techniques.

Unlike other methods, Slingshot does not restrict or require a priori knowledge of the number of lineages. The cluster-based minimum spanning tree enables the discovery of an arbitrary number of lineages, while also providing an intuitive framework for optional local supervision through the specification of the initial cluster and any number of terminal clusters. Lineage characteristics such as initial and terminal states can be difficult to identify at the level of individual cells due to drop-out effects and high levels of noise in single-cell data. This presents a challenge for methods based on robust cell-to-cell distances, such as Wishbone and DPT, the latter of which allows both beginning and endpoint specification at the level of individual cells. Conversely, more unsupervised methods, which only require an orientation for an otherwise unsupervised path, such as Monocle and Embeddr, can end up missing the initial state altogether. We find that local supervision at the cluster level provides a nice balance: due to averaging, clusters are less ambiguous than individual cells, making them easier to identify based on known marker genes, and specifying initial and terminal states provides an intuitive, but not overly restrictive way to ensure that inferred lineages are consistent with previously established results. Importantly, the (optional) specification of known terminal states is a form of local supervision that does not restrict the discovery of novel terminal states represented by other clusters. Furthermore, even in the case where all the terminal states are well characterized, the order and timing of branching events are often unknown and local supervision can enhance the inference of the global branching structure, as demonstrated in the “Results and discussion” section with the OE dataset of [26]. When there is no prior biological knowledge, Slingshot can be applied in an unsupervised manner as to the number and identity of terminal clusters.

It is also important to consider the amount of uncertainty in lineage and pseudotime inference, as this can impact downstream analyses such as the identification of differentially expressed genes along or between lineages, as noted in [30]. For branching lineage structures, there are two inextricably linked sources of uncertainty: the assignment of cells to lineages (“structural uncertainty”) and the calculation of each cell’s pseudotime (“temporal uncertainty”). While Slingshot only provides point estimates of lineage identities and pseudotimes, we note that it is generally computationally fast enough to be used in a bootstrap estimation procedure. Provided that the normalization, dimensionality reduction, and clustering steps are similarly fast and that any supervision provided by researchers can be automated, a bootstrap procedure could be used to assess uncertainty for the entire inference process.

Since there are many aspects to the problem of lineage inference, from sample collection to final analysis, it is important to define precisely the tasks for which Slingshot is designed. The philosophy of Slingshot is that upstream analysis steps such as normalization, dimensionality reduction, and clustering do not have a single solution that works well for all data types; these choices should therefore not be hardcoded into a lineage inference method. For example, Slingshot does not enforce a specific dimensionality reduction method because single-cell data can come from a variety of assays and in a wide range of dimensions, from the 271 cells×47,192 genes RNA-Seq dataset of [3] to the 25,000 cells×13 markers mass cytometry dataset of [9]. While the RGE method of Monocle 2 may work well in certain cases and the diffusion maps of Wishbone in others, this extreme heterogeneity seems to preclude any one-size-fits-all solution. Similar arguments can be made for other upstream analysis steps. Instead, Slingshot was designed with modularity in mind. Though it will typically come after normalization, dimensionality reduction, and clustering steps in an analysis pipeline, it is not a method for addressing these problems. For example, Slingshot led to biologically meaningful and novel results with PCA in [26] and with diffusion maps in [31]. Slingshot was applied using our recommended single-cell RNA-Seq data analysis workflow in [19]: the pipeline includes the data-adaptive selection of a normalization procedure (scone package; [20]), dimensionality reduction using a zero-inflated negative binomial model (zinbwave package; [21]), and resampling-based sequential ensemble clustering (RSEC; clusterExperiment package; [22]).

Ultimately, single-cell data are noisy, high-dimensional, and may contain a multitude of competing, interwoven signals. In the presence of such data, Slingshot provides a robust and modular method for lineage and pseudotime inference, that allows for novel lineage discovery, meaningful incorporation of biological constraints, and fits easily within existing analysis pipelines.

## Methods

We start from an *n*×*J* matrix of normalized expression measures (e.g., read counts) for *n* single cells and *J* genes or features. Slingshot assumes that the *n* cells have been partitioned into *K* clusters, potentially corresponding to distinct cellular states. Although Slingshot can in principle be applied directly to the normalized expression measures, we strongly recommend a dimensionality reduction step before pseudotemporal reconstruction, as Slingshot’s curve-fitting step uses Euclidean (or related) distances, which can misbehave in high-dimensional spaces (cf. curse of dimensionality). Dimensionality reduction can also strengthen signal in the data and help with visualization. We denote the dimension of the reduced space by *J*^′^.

Before detailing Slingshot’s two main steps, we introduce some notation. First, denote by *X*=(*X*_*ij*_) the *n*×*J*^′^ reduced-dimensional matrix of gene expression measures, for cells *i*∈{1,…,*n*} and dimensions *j*∈{1,…,*J*^′^}. Let $\left \{\mathcal {C}_{1},\ldots,\mathcal {C}_{K}\right \}$ denote the *K* cell clusters or states, i.e., disjoint subsets of cells, typically obtained by clustering the cells based on their gene expression measures. We then define a lineage as an ordered set of clusters and let *L* denote the total number of lineages. For a particular lineage, $\mathcal {L}_{l}$, denote its length (i.e., the number of clusters in the lineage) by *K*_*l*_ and the *k*^th^ cluster by $\mathcal {C}^{l}_{k}$, for *l*∈{1,…,*L*} and *k*∈{1,…,*K*_*l*_}. In particular, $\mathcal {C}^{l}_{1}$ and $\mathcal {C}^{l}_{K_{l}}$ correspond, respectively, to the initial and terminal states for the *l*^th^ lineage. It is important to note that a cluster can belong to multiple lineages and that the ordering of the clusters within a lineage does not strictly determine the final relative orderings of cells in those clusters.

As a given cluster can belong to multiple lineages, so can a cell. We therefore allow cells to have distinct pseudotime values for each lineage they are a part of. The pseudotime value for cell *i* in lineage *l* is denoted by $t^{l}_{i} \in \mathbb {R}_{\geq 0}$; if cell *i* does not belong to lineage *l*, i.e., $i \notin \cup _{k=1}^{K_{l}} \mathcal {C}^{l}_{k}$, then set $t^{l}_{i} = \emptyset $. The vector of pseudotime values for lineage *l* is denoted by $\mathbf {t}^{l} = \left (t^{l}_{i}: i=1,\ldots,n\right)$.

### Identification of cluster-based lineages

In its first step, Slingshot identifies lineages by treating clusters of cells as nodes in a graph and drawing a minimum spanning tree (MST) between the nodes, similar to the work of [7, 10]. Lineages are then defined as ordered sets of clusters created by tracing paths through the MST, starting from a given root node. Our method differs however in a number of important respects from those of [7, 10] including the distance measure used for drawing the tree and the (optional) incorporation of biologically meaningful supervision.

#### Shape-sensitive distance measure between cell clusters

Constructing an MST involves specifying a distance measure between nodes (in this case, cell clusters). We have found that a Mahalanobis-like distance, i.e., a covariance-scaled Euclidean distance, that accounts for cluster shape, works well in practice, but users have the option of specifying any type of distance measure (e.g., Euclidean, Manhattan). Specifically, the pairwise distance between clusters *i* and *j*, $d(\mathcal {C}_{i},\mathcal {C}_{j})$, is defined as

1 $$ d^{2}(\mathcal{C}_{i},\mathcal{C}_{j}) \equiv (\bar{X}_{i} - \bar{X}_{j})^{T} (S_{i} + S_{j})^{-1} (\bar{X}_{i} - \bar{X}_{j}),   $$

where $\bar {X}_{i}$ represents the center (mean) of cluster *i* and *S*_*i*_ its empirical covariance matrix in the reduced-dimensional space. This is essentially a multivariate *t*-statistic. By default, Slingshot uses the full covariance matrix of each cluster, allowing us to draw trees that are better covered by and representative of the cells in a dataset. However, in the presence of small clusters, the matrix *S*_*i*_+*S*_*j*_ may not be invertible and we may replace the full covariance matrix with the corresponding diagonal covariance matrix.

Some clustering algorithms return probabilities of cluster membership rather than hard assignments. In these cases, the cluster membership probabilities can be naturally and readily incorporated as weights in most distance measures. For instance, for the Mahalanobis-like distance of Eq. 1, we would compute weighted means and covariance matrices.

#### Biologically meaningful supervision

Slingshot allows two forms of supervision during lineage identification: initial state and terminal states specification. Like other methods (TSCAN, Waterfall, Monocle 2), Slingshot requires the user to identify the initial cluster or root node. Subsequently, every direct path from this node to a leaf node (i.e., a cluster with only one edge) will be called a lineage. Indeed, all existing lineage inference methods explicitly or implicitly make the assumption that a starting state can be identified by the user: Monocle and Embeddr construct orderings for which the user must select the correct direction and Wishbone and DPT require the user to select an initial cell or group of cells. In the simple case where the MST constructed by Slingshot has only two leaf nodes and one is specified as the root, this results in a single lineage. If an interior (non-leaf) node is specified as the origin, this results in two lineages, one terminating in each leaf node. Clusters with more than two edges will create bifurcations and produce additional lineages.

Additionally, Slingshot optionally allows the user to provide further supervision in the inference of the lineages by selecting clusters known to represent terminal cell states, imposing a local constraint on the MST algorithm. The constrained MST is obtained by first constructing the MST on all non-selected clusters and then connecting each selected cluster to its nearest non-selected neighbor. Such *local supervision* results in more biologically meaningful lineages for situations where the data can be explained by many possible lineage structures. Identified lineages are by construction consistent with known biology and provide improved stability over less supervised methods. Although terminal state supervision is not required, in many settings researchers do have knowledge of the cell types present in their data and systematically incorporating this knowledge can provide more accurate and stable inference. Importantly, this type of local supervision does not prevent the discovery of novel lineages; it allows the incorporation of specific knowledge of cell clusters, without imposing restrictions on the global branching structure. Ultimately, detecting multiple lineages based on gene expression data is a difficult problem that benefits from such guidance, as we demonstrate in the “Results and discussion” section.

### Identification of individual cell pseudotimes

The second stage of Slingshot is concerned with assigning pseudotimes to individual cells. For this purpose, we make use of principal curves [13] to draw a path through the gene expression space of each lineage. As we show in the “Results and discussion” section, principal curves give very robust pseudotimes when there is a single lineage. Multiple lineages demand more care and are handled using the simultaneous principal curves method proposed below. Indeed, just as clusters in the MST may belong to one or more lineages, the cells which constitute these clusters may be assigned to one or more lineages. In principle, we could construct standard principal curves for each lineage separately to arrive at pseudotimes. However, there is no guarantee that these curves would agree with each other in the neighborhood of clusters shared between lineages, so cells belonging to multiple lineages could be assigned very different pseudotime orderings by each curve. Since we assume a smooth differentiation process, this is potentially a violation and may be problematic in downstream analysis.

We therefore introduce a method of simultaneously fitting the principal curves of each lineage, which shrinks the curves to a consensus path in areas where lineages share many common cells, but allows the curves to separate as they share fewer and fewer cells. This ensures smooth bifurcations of the paths. We call the resulting curves *simultaneous principal curves*, as they are fit by an iterative procedure based on the principal curves algorithm of [13]. When there is only a single lineage (*L*=1), the pseudotimes of Slingshot are found by the standard principal curves algorithm, except that the initial curve is based on the lineage’s path through the MST found in the first stage (see below for details), rather than the first principal component. Additional file 1: Figure S19 illustrates the main steps in the simultaneous principal curves algorithm.

**Standard principal curves algorithm.** We first review the standard principal curves algorithm of [13] (for a single curve) in order to be clear about how we adapt it for simultaneous principal curves. After specification of an initial curve, the algorithm iteratively follows these steps:

- **1.**  Project all data points onto the curve and calculate the arc length from the beginning of the curve to each point’s projection. Setting the lowest value to zero, this produces pseudotimes.
- **2.**  For each dimension *j*, *j*∈{1,…,*J*^′^}, use the cells’ pseudotimes to predict their coordinates, typically with a smoothing spline. This produces a set of *J*^′^ functions which collectively map pseudotime values in $\mathbb {R}_{\geq 0}$ into $\mathbb {R}^{J'}$, thereby defining a smooth curve in *J*^′^ dimensions.
- **3.**  Repeat this process until convergence. We use the sum of squared distances between cells’ actual coordinates and their projections on the curves to determine convergence.

We note that these curves use the unit-speed parameterization, meaning that a principal curve defined by $\mathbf {c}(t):\mathbb {R}_{\geq 0} \rightarrow \mathbb {R}^{J^{\prime }}$ will satisfy ||**c**^′^(*t*)||=1 at all points *t* in the domain of **c**. This property ensures the equivalence between arc length and pseudotime mentioned in **Step 1**.

In order to characterize multiple branching lineages, we modify the iterative principal curves algorithm in two ways: by incorporating cell weights representing their assignment to particular lineages and by adding a shrinkage procedure to ensure smooth branching events. The *cell weights* are added in **Step 1**, with each cell’s weight for a given lineage being based on its projection distance to the curve representing that lineage. The *shrinkage* is performed in **Step 2**, by first recursively constructing an average curve for each branching event, then recursively shrinking the branching lineage curves toward this average. Thus, as with the individual lineage curves, each average curve is a function of pseudotime and can, itself, be averaged and shrunk.

In the case of branching lineages, where *L* is the total number of lineages (i.e., terminal states), our goal is to infer, for each lineage *l*∈{1,…,*L*}, a vector of pseudotime values, $\mathbf {t}^{l} = \left (t^{l}_{i}: i=1,\ldots,n\right)$, and a vector-valued function, $\mathbf {c}_{l}: \mathbb {R}_{\geq 0} \rightarrow \mathbb {R}^{J^{\prime }}$, for the associated curve in the low-dimensional space.

**Average curve construction for each branching event.** Average curves are constructed in a recursive manner, from the latest branching events to the earliest (i.e., from the leaves of the MST to the root). Consider a branching event involving *M* curves (typically *M*=2), $\mathbf {c}_{m}: \mathbb {R}_{\geq 0} \rightarrow \mathbb {R}^{J'}$, *m*=1,…,*M*, which may either be individual lineage curves (for branching events leading to leaf nodes) or average curves (for lineages differentiating in later branching events). The *average curve* is simply defined as

2 $$ \mathbf{c}_{\text{avg}}(t) \equiv \frac{1}{m} \sum_{m=1}^{M} \mathbf{c}_{m}(t),  $$

for values of *t* in the domains of each curve being averaged. Because all lineages share the same root cluster, we ensure that the starting point of all average curves (**c**_avg_(0)) will be identical, as will the starting point of the resulting shrunken curves. For branching events leading to leaf nodes, the curves being averaged will be individual lineage curves, whereas earlier branching events may also involve averaging average curves.

This recursive procedure ensures that the average curves constructed for early branching events are blind to the number of lineages ultimately produced by each branch. Without this condition, an early bifurcation event between a lineage that ends in a single terminal state and another that gives rise to multiple terminal states would produce an average curve that was strongly biased toward the latter branch.

**Shrinkage for each branching event.** Next, we perform shrinkage for each branching event, bringing the branching curves into better agreement in regions of shared cells. Unlike the construction of the average curves, this recursive process starts with the root and works out toward the leaves, meaning that the earliest branching event is the first to be shrunk. Let **c**_avg_ denote the average curve for a given branching event and let **c**_*m*_ denote one of the *M* curves to be shrunk at this event. Again, it may be the case that the curves being shrunk together at this step represent single lineages or averages of other curves. To determine how much each curve is shrunk toward the average, we construct curve-specific weighting functions $w_{m}: \mathbb {R}_{\geq 0} \rightarrow [0,1]$, as detailed below, with the constraint that *w*_*m*_ must be non-increasing. Additionally, by specifying that *w*_*m*_(0)=1 (representing the maximum amount of shrinkage), we ensure that diverging curves always share the same initial point. These weighting functions allow us to shrink the diverging curves toward the average curve with the additional update in **Step 2**:

3 $$ \mathbf{c}_{m}^{\text{new}}(t) \equiv w_{m}(t)\mathbf{c}_{\text{avg}}(t) + (1-w_{m}(t))\mathbf{c}_{m}(t).  $$

If all the weighting functions are smooth, this shrinkage step ensures that the final curves will follow a tree structure with smooth branching events.

**Weighting functions for each branching event.** Slingshot’s default weighting functions satisfy these conditions, i.e., are smooth, non-increasing, and take on a value of one at the origin. They are based on the distribution of pseudotimes for cells shared between the lineages corresponding to the branching curves. Specifically, for a branching event involving lineages {1,…,*H*}, we define a set of shared cells $\left \{i: t_{i}^{1} \neq \emptyset,\ldots, t_{i}^{H} \neq \emptyset \right \}$ and, for one of the *M* curves **c**_*m*_ to be shrunk at this event, we let $t_{min}^{m}$ and $t_{max}^{m}$ denote, respectively, the lowest and highest non-outlier pseudotimes for these cells along the curve (where outliers are defined by the 1.5 IQR rule of boxplots, where IQR stands for inter-quartile range). The *weighting function* for curve **c**_*m*_ is then defined as:

4 $$ w_{m}(t) \equiv \left\{\begin{array}{ll} 1, & 0 \leq t < t_{min}^{m} \\ 1 - F_{K}\left(\frac{t}{t_{max}^{m}-t_{min}^{m}}-\frac{1}{2}\right), & t_{min}^{m} \leq t \leq t_{max}^{m} \\ 0, & t > t_{max}^{m} \end{array}\right.,  $$

where *F*_*K*_ is the cumulative distribution function of a standard cosine kernel with a bandwidth of $\frac {1}{6}$ (which places weight only on values between $-\frac {1}{2}$ and $\frac {1}{2}$). The final curves are highly robust to the choice of kernel function (Additional file 1: Figure S20).

In both the single and branching lineage cases, final pseudotime values are derived from each point’s orthogonal projection onto the curves. In the latter case, assignment of cells to lineages is determined by cell weights, which are calculated in **Step 1** of the algorithm, using a cell’s projection distance to each lineage curve. Cell weights may be useful in downstream analyses, such as the identification of genes that are differentially expressed along and between lineages.

Cells belonging to multiple lineages will have multiple pseudotime values, but these values will agree quite well for cells positioned before the lineage bifurcation. This is because all curves share a common point of origin, **c**_*m*_(0), and the weighting functions *w*_*m*_, which determine the amount of shrinkage, assign unit weight to the origin (complete shrinkage) and decrease smoothly throughout the neighborhood of shared cells. Therefore, in the region prior to the bifurcation, shrinkage forces the curves to closely follow their average curve and the pseudotimes obtained by projection onto these curves will be highly similar. Additional file 1: Figure S19e shows the improved agreement between simultaneous principal curves as compared to separate, standard principal curves.

**Initialization of simultaneous principal curves algorithm.** As mentioned above, we initialize the algorithm using the MST from the first stage. Specifically, for each lineage, we start with the piecewise linear path through the centers of the clusters contained in the lineage (in contrast, standard principal curves are initialized by the first principal component over all cells). Starting with the path through the cluster centers allows us to leverage the prior knowledge that went into lineage identification as well as to improve the speed and stability of the algorithm, though in practice, the two procedures typically converge to very similar curves.

### Datasets

We demonstrate the performance of Slingshot by applying it to three previously published single-cell RNA-Seq datasets, each with a different number of terminal cell types.

**HSMM dataset.** The first dataset is a subset of the data used in [3], which assayed 271 human skeletal muscle myoblasts (HSMM) in order to study their development into mature myotubes. This is an example of data with only a single lineage. In their analysis, [3] identify a population of contaminating interstitial mesenchymal cells, which we omit from the dataset. This results in a sample of 212 cells believed to form a single, continuous developmental lineage. For our analysis, we used the cluster labels generated by Monocle as well as a set of labels obtained via *k*-means clustering for a range of values of *k*, and, as in the original paper, we represented the data in two dimensions obtained by ICA. The normalized data were downloaded from the NCBI GEO database (accession GSE52529).

**qNSC dataset.** The second dataset comes from [10], who assayed 132 hippocampal quiescent neural stem cells (qNSC) and their immediate progeny from adult mice, cells known to be involved in neurogenesis. Their goal was to assess cellular heterogeneity among this population and analyze continuous-time developmental dynamics. After removing a few outliers, their analysis focuses on 101 cells believed to represent the development of qNSCs into intermediate progenitor cells (IPC), a transitional state between qNSCs and mature neurons. However, they note an additional cluster of 23 cells branching off of this lineage, potentially representing an alternative terminal cell type. As in the original paper, we used the hierarchical clustering labels and the first two principal components as the reduced-dimensional space. Rather than focus solely on the IPC lineage though, we characterized the developmental trajectory of both proposed cell fates. The normalized data and code for preliminary analysis were downloaded from GEO (accession GSE71485).

**OE dataset.** The third dataset is that of [26], featuring 616 cells from the adult mouse olfactory epithelium (OE), tracing the development of quiescent stem cells into three unique terminal cell fates. The primary lineage maps the development of horizontal basal cells (HBC) into mature olfactory sensory neurons (mOSN) via a series of intermediate states. The secondary lineage gives rise to the support sustentacular (mSus) cells of this system and features fewer identifiable intermediates. A third lineage, which appears to split from the neuronal path, leads to a cluster of microvillous (MV) cells. Again, we relied on the cluster labels of the authors, who used a resampling-based sequential ensemble clustering (RSEC) approach [22], and represent cells by their coordinates along the first five principal components. The normalized data and cluster labels are available from GEO (accession GSE95601).

### Simulation study

**Simulation parameters.** In order to examine the performance of Slingshot and other methods in a wide range of scenarios, we performed a simulation study using the Bioconductor R package splatter [28] to produce artificial single-cell RNA-Seq datasets. Many parameters can potentially be tuned to generate these datasets, including parameters determining the distribution of mean gene expression, library size, outlier expression, drop-out, and the biological coefficient of variation. In order to make our simulation study as realistic as possible, we used a published dataset [3] to learn properties of the marginal distributions of the expression measures for both genes and samples.

In the first part of the study, simulated datasets consisted of two branching lineages (Fig. 4a). The number of cells *n* was varied from 120 to 1500, by increments of 60 cells. Additionally, we adjusted the signal-to-noise ratio by varying the probability of a gene being differentially expressed (DE) along a path between 0.1 (weak signal) and 0.5 (strong signal), by increments of 0.1. For each combination of sample size and DE proportion, we simulated 10 datasets, for a total of 1,200. In the second part, simulated datasets consisted of five branching lineages (Fig. 4c). The number of cells *n* was varied between 220 and 1,320, by increments of 220. The DE proportion was varied between 0.1 and 0.5, as in the two-lineage setting. Since all methods under consideration can accommodate non-linear paths, the probability of non-linear DE patterns was set to 0.5, meaning that half of all DE genes’ true average expression level varied according to a non-linear function of pseudotime.

**Clustering.** We examined Slingshot’s robustness to the choice of clustering method by performing hierarchical clustering, *k*-means clustering, and Gaussian mixture modeling (GMM), to obtain *K*=3 to 10 clusters on the three-dimensional representation of each simulated dataset obtained by PCA. Fixing the dimensionality reduction technique allows us to focus on the effects of the clustering method for the dimensionality reduction technique used. In order to alleviate the potential impact of outliers, whenever any method identified a cluster consisting of 4 cells or fewer, that cluster was removed and the method was re-run on the remaining cells.

For the purpose of comparing Slingshot with other lineage inference methods, we again used the top three principal components and set the clustering technique to be the Gaussian mixture model which minimizes the Bayesian information criterion (BIC). This is the default behavior of the mclust R package [32] and similar to the approach taken by TSCAN, which uses a variable number of principal components inferred from the data.

**Evaluation.** Methods were evaluated according to the agreement between inferred and true pseudotime variables for each lineage, as measured by the Kendall rank correlation coefficient. The Kendall rank correlation coefficient assesses the ordinal association between inferred pseudotimes and true pseudotimes, making it more robust to outliers and non-linearity than the Pearson correlation coefficient. We use a slight variant of this measure designed to reflect errors in the assignment of cells to lineages. Defining $\mathcal {S}_{0}$ as the set of cells along a true lineage and $\mathcal {S}_{1}$ as the set of cells along an inferred lineage, we calculate:

5 $$ \tau \equiv \frac{(\text{\# of concordant pairs})-(\text{\# of discordant pairs})}{{|\mathcal{S}_{0} \cup \mathcal{S}_{1}| \choose 2}},  $$

where concordant and discordant pairs are defined strictly, not allowing for ties. Hence, only cells belonging to both the true and inferred lineages (i.e., in $\mathcal {S}_{0} \cap \mathcal {S}_{1}$) contribute to the numerator. Cells which are along the true lineage (i.e., elements of $\mathcal {S}_{0}$) and not assigned a pseudotime by the inferred lineage (not in $\mathcal {S}_{1}$) will only contribute to the denominator, bringing *τ* closer to 0. Similarly for extraneous cells which are included in $\mathcal {S}_{1}$ but not in $\mathcal {S}_{0}$.

For each true lineage, we take the maximum *τ* over all inferred lineages and average these values within a single dataset. This produces a bias in favor of methods that identify many, potentially spurious lineages, as there will be more values over which to take the maximum. We do not correct for this bias, but simply note that Monocle 2 and DPT-Full are the methods which seem to benefit the most from it.

## Additional file

Additional file 1 Supplemental methods for the analysis of the olfactory epithelium data and supplemental figures 1-20. (ZIP 34910 kb)

## Abbreviations

BIC: Bayesian information criterion
DDRTree: Discriminative dimensionality reduction via learning a tree
DE: Differential expression
DPT: Diffusion pseudotime
GBC: Globose basal cell
GEO: Gene expression omnibus
GPLVM: Gaussian process latent variable model
GMM: Gaussian mixture modeling
HBC: Horizontal basal cell
HSMM: Human skeletal muscle myoblast
ICA: Independent component analysis
IPC: Intermediate progenitor cell
*k*NN: k nearest neighbors
MCMC: Markov chain Monte-Carlo
MFA: Mixture of factor analysers
mOSN: Mature olfactory sensory neuron
MST: Minimum spanning tree
mSus: Mature sustentacular
MV: Microvillous
NCBI: National center for biotechnology information
OE: Olfactory epithelium
OMGP: Overlapping mixtures of Gaussian processes
PCA: Principal component analysis
qNSC: quiescent neural stem cells
RGE: Reverse graph embedding
RNA-Seq: Ribonucleic acid sequencing
RSEC: Resampling-based sequential ensemble clustering
t-SNE: t-distributed stochastic neighbor embedding
TSCAN: Tools for Single-Cell ANalysis
