# Supplementary material for: Slingshot: cell lineage and pseudotime inference for single-cell transcriptomics
Source: BMC Genomics. 2018 Jun 19;19:477. doi: 10.1186/s12864-018-4772-0 (PMC6007078; doi:10.1186/s12864-018-4772-0)
Supplement: Supplementary file 1 — Supplemental methods for the analysis of the olfactory epithelium data and supplemental figures 1-20. (ZIP 34910 kb) [file 12864_2018_4772_MOESM1_ESM.zip › slingshot-supplement.pdf]

1 Preprocessing of Olfactory Epithelium Dataset

Below we describe the upstream computational methods used in the analysis of the olfactory epithelium (OE) dataset of [1].

Normalization. After read mapping and the computation of gene-level counts, a typical RNA-Seq analysis involves a number of preprocessing steps, such as gene and sample filtering, log-transformation, and normalization. Raw gene expression profiles need to be normalized prior to lineage reconstruction to remove unwanted technical effects (e.g., batch effects) that bias expression measures [2].

We used the R package scone to apply and compare various normalization methods and select the one that performs best on the OE data, according to a pre-defined set of nine performance measures [3]. The selected normalization technique consisted of full-quantile normalization followed by regression of log-transformed read counts on the first principal component of a matrix of sample-level quality control measures unrelated to biological condition. The regression residuals were then shifted to have the same mean as the log-counts before the regression, yielding the log-scale normalized counts.

Clustering. Using (unsupervised) clustering to find biologically distinct classes of cells is another feature of many single-cell RNA-Seq data analysis pipelines. Clustering cells provides stability in downstream analysis by dramatically reducing the number of possible relationships between cells, reducing noise through averaging, and lessening the potential impact of outliers on the final ordering, as noted in [4] in the context of lineage inference.

The olfactory epithelium data were clustered with the `clusterMany` function from the Bioconductor R package clusterExperiment, which implements a resampling-based sequential ensemble clustering (RSEC) method to find tight, stable clusters [5]. Clustering was performed on the top 50 principal components using hierarchical clustering as the base method. To increase the stability of the clustering and lineage inference processes, a small percentage of cells may be left unclustered. Slingshot can then assign pseudotimes to these cells, after constructing simultaneous principal curves, by projecting them onto the nearest curve.

Supervision. Figure S1 displays the result of running Slingshot with and without supervision on the OE dataset. For this dataset, clusters corresponding to the neuronal, sustentacular, and microvillous cell types were identified based on known marker genes [1]. These cell types are known to be non-differentiating and should thus be the terminal states of their respective lineages. Without supervision (Figure S1a, the sustentacular cluster was not identified as an endpoint, yielding

results inconsistent with prior knowledge. Selecting the sustentacular cluster as an endpoint led to the recovery of the three lineages reported and validated in the original paper (Figure S1b).

2 Additional Results on the Olfactory Epithelium Dataset

In addition to Slingshot and Monocle 2, we applied several other methods to the three-lineage dataset of [1]. For visualization of all results, see Figures S3 and S4.

Monocle requires that the number of lineages be pre-specified, but in practice this number is unknown. Even with the proper number of lineages, Monocle's results contradicted prior knowledge, as it identified a spurious branching event along the neuronal lineage terminating in a cluster of GBCs, a known intermediate state (Figure S3e).

Diffusion pseudotime (DPT) requires the selection of an initial cell, for which we used the cell assigned the lowest pseudotime by Slingshot. And while DPT does not explicitly require knowledge of the number of lineages, we found that without supervision, it produced an overabundance of endpoints (Figure S3j). Thus, we also tried strategies where the number of lineages was limited to two or three (Figures S3h and S3i); in both cases DPT successfully identified a neuronal and sustentacular lineage, but incorrectly assigned microvillous cells to the former.

TSCAN was run as is, both with and without preprocessing (Figures S3g and S3f). Without preprocessing, two lineages were identified, both of which contained two known endpoints, sustentacular and microvillous cells. Additionally, the shorter lineage terminated in a cluster of GBCs, a known intermediate state. Two lineages were again identified when TSCAN was run with preprocessing. These appear to represent the microvillous and neuronal lineages, but both contain a fair number of sustentacular cells, as well.

Finally, since Wishbone can only handle a maximum of two lineages, we applied it to a subset of the data believed to represent the sustentacular and neuronal lineages (Figures S4a and S5a). Wishbone identified a bifurcation, but not one which separated neurons from sustentacular cells (Figures S4c and S5b). The large skip in the longer lineage seems to be an artifact of the diffusion map used for dimensionality reduction. When we used a modified version of Wishbone with principal component analysis as the dimensionality reduction step, we observed that this gap was no longer present, but Wishbone still failed to identify the primary bifurcation in the data (Figures S4d and S5b).

3 Simulation Study Design and Results

Slingshot's Robustness to Dimensionality Reduction. In order to assess Slingshot's robustness to the choice of dimensionality reduction technique, we ran each of the simulated two-

lineage datasets through four popular methods: Principal component analysis (PCA), independent component analysis (ICA), t-distributed stochastic neighbor embedding (t-SNE), and diffusion maps, using 2 to 8 dimensions for each. Before applying Slingshot, we clustered the resulting low-dimensional representation using Gaussian mixture modeling with the BIC criterion, as described in Methods and implemented in the mclust package [6].

For a qualitative comparison of these dimensionality reduction methods, as well as the method of reverse graph embedding (RGE) used by Monocle 2, see Figure S7. The distributions of accuracy scores generated by Slingshot with each dimensionality reduction technique are presented in Figure S8 and the relationships between these scores and the simulation parameters are displayed in Figure S9. We see similar patterns in PCA, ICA, and diffusion maps: In two dimensions, these methods produce a distinctly bimodal distribution of accuracy scores; in three dimensions, they produce a wide, flat distribution of scores; in four, we see consistently high scores, which tend to deteriorate slowly as more dimensions are added. The exception to this pattern is t-SNE, which produced consistently high accuracy scores, regardless of the number of dimensions used.

As with the choice of clustering method, there appears to be a range of possible dimensionality reduction techniques over which Slingshot is largely robust. We see this in the similar distributions of accuracy scores generated for four or more dimensions (Figure S8). However, as with clustering, there are still important differences both between methods and even within a given method. The two- and three-dimensional versions of PCA, ICA, and diffusion maps yield very different distributions of accuracy scores, suggesting that they may miss important signal beyond the first three dimensions. The same was not true of t-SNE, which showed impressive accuracy and stability over the full range of dimensionalities. This unexpected result is perhaps due to the "well-behaved" nature of the synthetic data, though it is noteworthy that t-SNE was the only method examined which showed more sensitivity to sample size than amount of signal (Figure S9). Overall, though, the variation in Slingshot's accuracy across dimensionality reduction methods is smaller than the variation in accuracy observed when comparing different lineage inference methods with a common dimensionality reduction step (Figure S10).

Monocle and Monocle 2. Both Monocle and Monocle 2 strongly recommend an initial gene filtering step, based on differential expression between timepoints. Since the simulated datasets did not contain different timepoints, we used two alternative techniques, one of which is suggested in the Monocle vignette. Specifically, starting from an initial set of $12,664$ genes, the 100 genes with the highest loadings on each of the top p principal components were selected, generally totaling close to $100p$ genes. This choice should ensure some similarity between the Monocle results and those of Slingshot and TSCAN, both of which use PCA for dimensionality reduction. The number

of PCs from which to take the top 100 genes was determined by the eventual size of the reduced-dimensional space, J' (i.e., when using ICA to go down to three dimensions, we used the top 100 genes from the top three PCs). We refer to this method as "PC Loadings" in Figures S10 and S12. The second gene filtering technique uses the union of the $5,000$ genes with the highest average log-expression and the $5,000$ genes with the highest variance in log-expression, after full-quantile normalization. This technique was designed to be comparable to using all genes and typically retained around $6,000$ genes (for the four "extreme cases" shown in Figure S7, the number of genes retained were $6,096$, $6,096$, $6,051$, and $5,903$). With either version of Monocle, using all genes would have represented a significant computational cost. This method is referred to as "5K Mean/Var" in Figures S10 and S12.

For Monocle, the dimensionality reduction method used was ICA, with variable number of dimensions. For the two-lineage topology, we tried $J' = 2$ through 5 and for the five-lineage topology, we tried $J' = 3$ through 5. Across all combinations of gene sets and dimensionalities, the best set of results (by highest median accuracy score) is chosen to represent Monocle in Figure 4 and these results are compared to Slingshot results using the same dimensionality reduction technique. Knowledge of the true starting cell was used to orient pseudotime (via the "reverse" argument), such that the initial cell was always nearer to the beginning than the end of pseudotime. Since Monocle does not detect branching events, the correct number of lineages (2 or 5) was also provided. It was not clear why Monocle returned errors more often than any other method, but this appears to have been related to sample size, with larger samples producing errors more frequently.

For Monocle 2, dimensionality reduction was performed with DDRTree, again with $J' = 2$ through 5 in the two-lineage topology and $J' = 3$ through 5 in the five-lineage case. The results of Monocle 2 with the best performing gene set and J' are shown in Figure 4. After an initial ordering, the state containing the true initial cell was designated as the starting state and the cells were re-ordered. If this state was interior to the tree (i.e., did not contain an endpoint of a lineage), this would lead to an error, which we had to resolve. In these cases, lineages were identified as they are by Slingshot: As every unique path through the tree from the initial state to a terminal state. Even in datasets for which the initial cell was in a leaf state, Monocle 2 does not include an automated method for isolating individual paths, so one had to be produced. To do this, we modified code from the `BEAM` method, which analyzes branching events by examining the two lineages produced, to instead extract only one lineage.

Diffusion Pseudotime (DPT). Ordering by diffusion pseudotime (DPT) followed the process laid out in the vignette for the Bioconductor R package `destiny`. This consisted of dimensionality reduction by diffusion maps and ordering with the DPT algorithm, producing a single pseudotime

variable and a number of nested branch identities defining bifurcation events. However, as these branch identities often contained missing values (`NA`s), it was unclear how to fairly assess DPT's identification of branching events.

We therefore employed multiple strategies, the simplest being the one lineage strategy (or "DPT-1"), which ignored all branching results and treated the output pseudotime variable as a single lineage. Other numbered strategies started from the highest-level split and chose subsequent branching events iteratively from within the largest remaining branch until the predetermined number of lineages was reached. These strategies are thereby supervised in much the same way as Monocle. For both the two- and five-lineage topologies, we implemented strategies that used the correct number of lineages, as well as one lineage too few and one too many. Finally, a "Full" strategy which uses all branching events was also implemented. Because DPT uses twenty-dimensional diffusion maps, we compared it to the Slingshot implementation with eight-dimensional diffusion maps (the highest dimensionality for which we ran Slingshot).

As noted, the best method chosen for DPT in both topologies was the one-lineage strategy. We believe this is due to the large number of missing values present in the output branch identities. On average, in the two-lineage topology, 44.1% of cells were assigned a value of `NA` for the highest-level branching event. There was no clear relationship between this percentage and the number of cells present in a simulated dataset, though the distribution did get somewhat less variable with increased signal in the dataset (Figure S13).

TSCAN. TSCAN ordering was performed as shown in the vignette, with the `exprmclust` and `TSCANorder` functions from the Bioconductor R package `TSCAN`. Pseudotime variables were taken from the output with `orderonly` set to `FALSE`. TSCAN also provides its own preprocessing functionality, but applying this to the simulated data was seemingly detrimental. For both the two- and five-lineage topologies, running TSCAN with its own preprocessing, rather than full-quantile normalization, produced poor accuracy scores which tended to cluster around 0. Running this preprocessing after full-quantile normalization improved the performance modestly, but in both cases, the implementation with no preprocessing step performed the best.

The hybrid method, which uses TSCAN dimensionality reduction and clustering, was implemented with full-quantile normalization and no preprocessing step. The resulting clusters and low-dimensional data representation were then passed to Slingshot. Although TSCAN includes a cluster-based MST, the hybrid method uses Slingshot's version of this step.

Figure S1: *Impact of local supervision and distance measure on cluster-based MST: OE dataset.* We show three methods of constructing an MST on the clusters of the mouse olfactory epithelium dataset from [1]. Although we only visualize the first three principal components, we note that these trees were constructed in the five-dimensional space defined by the first five PCs. **Panel (a)**: Without endpoint supervision, we draw the (known) false conclusion that sustentacular cells may develop into GBCs (red segment). **Panel (c)**: Using Euclidean distances between cluster centers leads to the detection of a spurious branching event and the same erroneous conclusion about sustentacular cells (red segments). **Panel (b)**: Both of these issues are resolved by using Slingshot's covariance-scaled distance measure and local supervision to mark the mature sustentacular cell cluster as an endpoint. This final tree is consistent with known biology and branching events were validated in follow-up experiments. The starting cluster (HBC) is indicated in green and the terminal clusters (mOSN, mSus, and MV) are indicated in red.

Figure S2: *Slingshot: HSMM and qNSC datasets.* We validated Slingshot by applying it to previously analyzed datasets and comparing its results to published results. For the HSMM dataset of [7], we obtained a two-dimensional representation of the data by ICA, as in the original paper. We clustered the cells by k-means with $k = 2$. This allowed us to set the direction of the curve by specifying a starting cluster, similar to setting the direction of the path for Monocle. For the qNSC dataset of [8], we followed the original analysis, obtaining a two-dimensional representation of the data by PCA and cluster labels by hierarchical clustering on the counts matrix. Cells are color-coded by cluster, with the initial cell in red and blue, respectively, for the HSMM and qNSC datasets.

Figure S3: *Multiple lineage inference: OE dataset.* Pseudotime variables for each lineage inferred by several methods on the OE dataset of [1], along with known biological relationships between cell types. See Section 2 for details.

Figure S4: *Multiple lineage inference: Two-lineage subset of OE dataset.* Pseudotime variables for each lineage inferred by Slingshot and Wishbone on a subset of the OE dataset of [1] chosen to represent only the neuronal and sustentacular lineages (Wishbone can only identify a maximum of two lineages). **Panel (a)**: Known biological relationships between cell types. **Panel (b)**: Slingshot was run on the top three PCs with clusters identified by RSEC and the HBC cluster selected as the initial state. **Panel (c)**: Wishbone used the top four non-constant diffusion components for dimensionality reduction (as well as 3 and 5, not shown), along with 150 waypoints and $k = 30$ nearest neighbors for ordering. **Panel (d)**: Due to the highly irregular pseudotime variables produced by the methods in **Panel (c)**, we also implemented a modified version of Wishbone using the top 3 PCs for dimensionality reduction. However, this method also failed to identify the bifurcation between the sustentacular and neuronal lineages.

Figure S5: *Slingshot and Wishbone: Two-lineage subset of OE dataset.* In order to compare Slingshot and Wishbone, we took a subset of the OE dataset of [1] chosen to represent only the neuronal and sustentacular lineages (Wishbone can only identify a maximum of two lineages). Using PCA, Slingshot recovers the branching pattern originally discovered in the full dataset. Using its default dimensionality reduction method, diffusion eigenvectors, Wishbone does not capture the sustentacular lineage, but instead identifies a spurious branching event. Using PCA, Wishbone again fails to identify the sustentacular lineage and instead identifies a different spurious branching event.

Figure S6: *Simulation study: Parameter values for splatter package.* Two-lineage and five-lineage datasets were simulated using the function `splatSimulate` from the Bioconductor R package splatter, according to the parameters in the tables. Values highlighted in red were varied in the simulation. For the two-lineage topology, the number of cells varied between 120 and $1,500$, always with equal numbers of cells in each group. For the five-lineage topology, the number of cells varied between 220 and $1,320$, with the number of cells per group proportional to the length of the corresponding path. For both topologies, the probability of a gene being differentially expressed varied between 10% and 50%. Parameters not related to the topology were learned from the dataset of [7], after an initial filtering step requiring a gene to have at least 10 reads (the median of all non-zero counts) in at least 28 samples (10% of all samples).

Figure S7: *Impact of dimensionality reduction method: Simulated two-lineage datasets.* Dimensionality reduction is an important preprocessing step for lineage inference, as different methods can lead to highly divergent conclusions. We show how five different dimensionality reduction methods represent extreme scenarios of our simulation model (in terms of sample size and signal-to-noise), in the case of two branching lineages. For all methods, the two panels on the left correspond to the smallest signal-to-noise ratios while those on the right have the highest. Similarly, the top two panels correspond to the smallest number of cells ($n = 120$) while those on the bottom have the largest ($n = 1,500$). Color denotes the true pseudotime along the two lineages, with purple representing early, undifferentiated cells and green representing late, terminal cell fates. In each panel, the red cell is the true starting cell and the black cells are true terminal cells. See Section 3 for details on simulation scenarios.

Figure S8: *Robustness of Slingshot pseudotimes to dimensionality reduction: Simulated two-lineage datasets.* Gaussian kernel density plots of accuracy scores for different dimensionality reduction methods (columns) and numbers of dimensions (rows) based on simulated data with two lineages. After dimensionality reduction, clustering was performed by Gaussian mixture modeling with the BIC criterion. Slingshot's performance varies with the choice of dimensionality reduction method, however it is quite robust to the choice of the number of dimensions. In general, the average accuracy of inferred lineages improves slightly with increasing dimensionality, but this effect is small. See Section for the definition of accuracy scores based on Kendall's rank correlation coefficient and Section 3 for details on simulation scenarios.

Figure S9: *Slingshot accuracy scores by sample size and amount of signal, for four dimensionality reduction methods: Simulated two-lineage datasets.* For each of the four dimensionality reduction techniques shown in Figure S8, we examined the effect of sample size and signal-to-noise ratio on the accuracy of Slingshot's inferred pseudotimes. All implementations used Gaussian mixture modeling with the BIC criterion for clustering. Each cell in the pseudocolor images represents the average accuracy score over 30 simulated datasets at a given proportion of differentially expressed genes and sample size. With small sample size and low signal, Slingshot performs poorly, regardless of dimensionality reduction technique. With increased sample size and signal, the performance generally improves, but PCA, ICA, and diffusion maps appear more sensitive to changes in signal, while t-SNE appears more sensitive to changes in sample size. See Section for the definition of accuracy scores based on Kendall's rank correlation coefficient and Section 3 for details on simulation scenarios. Images were produced by the R package superheat [9].

Figure S10: *Comparison of accuracy scores for all lineage and pseudotime inference methods: Simulated two-lineage datasets.* Gaussian kernel density plots of accuracy scores show how different lineage and pseudotime inference methods performed on a series of simulated datasets with two terminal cell fates. Bars to the left of each density plot represent the percentage of datasets on which a method returned an error. Errors are treated as 0 values for calculating the median score, but are not included in the density estimates. See Section for the definition of accuracy scores based on Kendall's rank correlation coefficient and Section 3 for details on simulation scenarios.

Figure S11: *Accuracy scores by sample size and amount of signal, for all top lineage inference strategies: Simulated two-lineage datasets.* For each of the methods shown in Figure 4b, we examined the effect of sample size and signal-to-noise ratio on the accuracy of inferred pseudotimes. Each cell in the pseudocolor images represents the average accuracy score over 30 simulated datasets at a given proportion of differentially expressed genes and sample size. When calculating these averages, a score of zero was assigned any time a method returned an error. See Section for the definition of accuracy scores based on Kendall's rank correlation coefficient and Section 3 for details on simulation scenarios.

Figure S12: *Comparison of accuracy scores for all lineage and pseudotime inference methods: Simulated five-linage datasets.* Gaussian kernel density plots of accuracy scores show how different lineage and pseudotime inference methods performed on a series of simulated datasets with five terminal cell fates. Bars to the left of each density plot represent the percentage of datasets on which a method returned an error. Errors are treated as 0 values for calculating the median score, but are not included in the density estimates. See Section for the definition of accuracy scores based on Kendall's rank correlation coefficient and Section 3 for details on simulation scenarios.

Figure S13: *Missing branch identities in DPT: Simulated two-lineage datasets.* For each simulated dataset in the two-lineage setting, we recorded the percentage of cells that were assigned a value of `NA` for the highest-level branching event by DPT. **Left:** Boxplots depict the distributions of these percentages at all sample sizes. **Right:** Boxplots depict the distributions of these percentages at all levels of signal. See Section 3 for details on simulation scenarios.

Figure S14: *Number of inferred lineages by sample size: Simulated two-lineage datasets.* The numbers of lineages produced by Slingshot and TSCAN are robust to the number of cells, n. By contrast, the number of lineages inferred by Monocle 2 increases with sample size. Results correspond to the strategies shown in Figure 4b, with the Slingshot implementation using two-dimensional ICA and GMM, but similar patterns hold for other scenarios. Note the difference in y-axis scales. See Section 3 for details on simulation scenarios.

Figure S15: *Comparison of accuracy scores for lineage and pseudotime inference methods: Simulated five-lineage datasets.* **Panel (a)**: The five-lineage structure contains three distinct types of lineages: Lineages 1 and 2 consist of short, long, and short segments (in order); Lineage 3 consists of short, short, and long segments; and Lineages 4 and 5 consist of four short segments. **Panel (b)**: Gaussian kernel density plots of accuracy scores show that Slingshot, TSCAN, and Monocle 2 were all able to characterize Lineage 3 more accurately than the other lineages. Bars to the left of each density plot represent the percentage of datasets on which a method returned an error. Errors are treated as 0 values for calculating the median score, but are not included in the density estimates. See Section for the definition of accuracy scores based on Kendall's rank correlation coefficient and Section 3 for details on simulation scenarios.

Figure S16: *Robustness of cluster-based MST: HSMM dataset.* Using the single-lineage HSMM dataset of [7], we show that the cluster-based MST method (similar to TSCAN and Waterfall), using k-means clustering, is fairly robust to noise for a range of values for the number of clusters k, not only for $k = 5$, as shown in the main text (Figure 2). As in [7], dimensionality reduction is performed by ICA. We examine the stability of the method by plotting pseudotimes based on 50 subsamples of the data vs. the original pseudotimes. Subsamples were generated in a bootstrap-like manner, by randomly sampling n times, with replacement from the original cell-level data and retaining only one instance of each cell. Thus, subsamples were of variable sizes, but contained on average about 63% of the original cells. The cluster-based MST method occasionally detected spurious branching events and, for the purpose of visualization, cells not placed along the main lineage were assigned a pseudotime value of 0.

Figure S17: *Robustness to clustering of Slingshot and cluster-based MST (TSCAN and Waterfall): Simulated two-lineage datasets.* **Panel (a)**: Using a simulated two-dimensional dataset with two lineages, k-means to assign cluster labels ($k = 3, \ldots, 14$), and Slingshot's covariance-scaled distance measure, we show that the cluster-based MST (as in TSCAN and Waterfall) can be highly variable, even while identifying the correct global structure (purple). Despite using these same trees, Slingshot's simultaneous principal curves are robust to this variability and all of them produce nearly identical results (green). **Panel (b)**: With the same dataset as above, we show two pathological scenarios ($k = 2$ and $k = 15$) in which the cluster-based MST fails to identify the correct global structure (purple); the corresponding simultaneous principal curves are plotted in green. For $k = 2$, the MST is unable to detect a branching event and the resulting curve attempts to fit all of the data. With higher numbers of clusters, we eventually run into the problem of overfitting and the MST detects spurious branching events. Thus, for $k = 15$, the corresponding simultaneous principal curves similarly overfit certain regions of the data.

Figure S18: *Difference in accuracy between TSCAN and hybrid method: Simulated datasets.* Gaussian kernel density plots of accuracy scores for TSCAN (red) and a hybrid method (green, with red lines) and differences in accuracy scores between the hybrid and TSCAN (Hybrid-TSCAN, green). The hybrid method uses TSCAN for dimensionality reduction and clustering, then Slingshot for building the cluster-based MST and inferring pseudotimes. Slingshot's shape-sensitive cluster distance measure and simultaneous principal curves tend to improve the accuracy of inferred pseudotimes over TSCAN alone. In the two-lineage setting, the hybrid method improved upon TSCAN's accuracy in 79.2% of datasets and in the five-lineage setting, 66%. See Section for the definition of accuracy scores based on Kendall's rank correlation coefficient and Section 3 for details on simulation scenarios.

(a) *Constructing initial curves using a cluster-based MST.* Using a synthetic, two-dimensional dataset with two lineages of different lengths, we show how Slingshot constructs the initial curves from the MST and the corresponding pseudotime variables by orthogonal projection. Clusters were obtained by k-means and orthogonal projection lines are shaded to represent cell-level weighting (for cells belonging to both lineages, the weights are proportional to the ratio of projection distances to each curve; at this stage, weighting only affects cells from the green cluster).

(b) *Updating the curves with a scatterplot smoother.* For each curve, we use a smoothing spline to infer the cells' coordinates in each dimension as a function of pseudotime. The resulting functions define a curve in the original, low-dimensional space.

(c) *Average curves and weighting functions.* The average curve, $\mathbf{c}_{avg}(t)$, is the pointwise average of the two curves $\mathbf{c}_1(t)$ and $\mathbf{c}_2(t)$, defined for all t in the intersection of their domains. Weighting functions are constructed for each curve based on the pseudotime distribution of shared cells (cells belonging to all lineages involved in the branching event). The upper and lower whiskers of the boxplot define the boundaries of the decreasing part of the weighting function (by default, a scaled version of the survival function of the cosine kernel).

(d) *Effect of shrinkage over one iteration.* Dashed lines show the unshrunken curves from above, solid lines show the new, shrunken curves. Over a single iteration, the shrinkage step has a very small effect, primarily in the neighborhood of cells common to both lineages.

(e) *Effect of shrinkage over entire algorithm.* Individual principal curves will tend to disagree in the neighborhood of shared cells, where they should be similar. Shrinkage reduces the discrepancy between curves in this neighborhood, but has little effect beyond it.

Figure S19: *Simultaneous principal curves: Detailed explanation of the shrinkage step.* Using a toy dataset generated by adding Gaussian noise to two two-dimensional smooth, branching curves, we illustrate the impact of the shrinkage step in simultaneous principal curves.

Figure S20: *Simultaneous principal curves: Robustness to weighting functions.* **Panel (a)**: Using the synthetic dataset shown in Figure S19 with two lineages of different lengths, Slingshot outputs very similar curves regardless of the choice of kernel used for producing weighting functions when performing shrinkage. **Panel (b)**: Slingshot pseudotimes are very robust to the choice of kernel, as indicated by the very high average Pearson correlation coefficients between pseudotimes for pairs of comparable lineages (i.e., the longer lineage paired with the longer lineage and the shorter lineage with the shorter lineage).

4 Supplemental Figure Captions

References

[1] R. B. Fletcher, D. Das, L. Gadye, K. Street, A. Baudhuin, D. Risso, A. Wagner, M. B. Cole, Q. Flores, Y. G. Choi, N. Yosef, E. Purdom, S. Dudoit, and J. Ngai, "Deconstructing Olfactory Stem Cell Trajectories at Single-Cell Resolution," *Cell Stem Cell*, vol. 20, pp. 817–830, May 2017.

[2] C. A. Vallejos, D. Risso, A. Scialdone, S. Dudoit, and J. C. Marioni, "Normalizing single-cell RNA sequencing data: challenges and opportunities," *Nature Methods*, vol. 14, pp. 565–571, June 2017.

[3] M. Cole and D. Risso, *scone: Single Cell Overview of Normalized Expression data*, 2017. R package version 1.1.2.

[4] Z. Ji and H. Ji, "TSCAN: Pseudo-time reconstruction and evaluation in single-cell RNA-seq analysis," *Nucleic Acids Research*, vol. 44, p. e117, July 2016.

[5] E. Purdom and D. Risso, *clusterExperiment: Compare Clusterings for Single-Cell Sequencing*, 2017. R package version 1.3.3.

[6] C. Fraley, A. E. Raftery, T. B. Murphy, and L. Scrucca, *mclust Version 4 for R: Normal Mixture Modeling for Model-Based Clustering, Classification, and Density Estimation*, 2012.

[7] C. Trapnell, D. Cacchiarelli, J. Grimsby, P. Pokharel, S. Li, M. Morse, N. J. Lennon, K. J. Livak, T. S. Mikkelsen, and J. L. Rinn, "The dynamics and regulators of cell fate decisions are revealed by pseudotemporal ordering of single cells," *Nature Biotechnology*, vol. 4, no. 32, pp. 381–391, 2014.

[8] J. Shin, D. A. Berg, Y. Zhu, J. Y. Shin, J. Song, M. A. Bonaguidi, G. Enikolopov, D. W. Nauen, K. M. Christian, G. Ming, and H. Song, "Single-cell RNA-Seq with Waterfall reveals molecular cascades underlying adult neurogenesis," *Cell Stem Cell*, vol. 17, no. 3, pp. 360–372, 2015.

[9] R. Barter and B. Yu, *superheat: A Graphical Tool for Exploring Complex Datasets Using Heatmaps*. R package version 0.1.0.
