# Supplementary material for: Slingshot: cell lineage and pseudotime inference for single-cell transcriptomics
Source: BMC Genomics. 2018 Jun 19;19:477. doi: 10.1186/s12864-018-4772-0 (PMC6007078; doi:10.1186/s12864-018-4772-0)
Supplement: Supplementary file 1 — Supplemental methods for the analysis of the olfactory epithelium data and supplemental figures 1-20. (ZIP 34910 kb) [file 12864_2018_4772_MOESM1_ESM.zip › FIGURE-S6.pdf]

a

Global:

Genes	Cells	Seed
8157	120	1 - 1200

Groups:

Groups	Group Cells
3	40, 40, 40

Mean:

Rate	Shape
0.052077081	1.15659587

Library size:

Location	Scale
13.09876617	0.184200917

Exprs outliers:

Probability	Location	Scale
0.069020473	3.22019918	0.872743773

Diff expr:

Probability	Down Prob	Location	Scale
0.1	0.5	0.1	0.4

BCV:

Common Disp	DOF
0.983853072	11.07317421

Dropout:

Present	Midpoint	Shape
FALSE	2.173935299	-0.995546968

Paths:

From	Length	Skew	Non-linear	Sigma Factor
0, 1, 1	1000	0.5	0.5	0.8

b

Global:

Genes	Cells	Seed
12664	220	1 - 300

Batches:

Batches	Batch Cells	Location	Scale
1	220	0.1	0.1

Mean:

Rate	Shape
0.011465886	0.870938558

Library size:

Location	Scale
14.3675295	0.63163865

Exprs outliers:

Probability	Location	Scale
0.034112445	3.471323036	0.70410172

Groups:

Groups	Group Probs
11	(1,2,1,1,1,1,2,1,1)/11

Diff expr:

Probability	Down Prob	Location	Scale
0.1	0.5	0.25	1

BCV:

Common Disp	DOF
0.937394291	9.61882708

Dropout:

Present	Midpoint	Shape
TRUE	1.968930841	-0.5888576

Paths:

From	Length	Skew	Non-linear	Sigma Factor
0,1,2,2,1,5,5,6,6	(1,2,1,1,1,1,2,1,1)*100	0.5	0.5	0.8
