# Supplementary material for: Slingshot: cell lineage and pseudotime inference for single-cell transcriptomics
Source: BMC Genomics. 2018 Jun 19;19:477. doi: 10.1186/s12864-018-4772-0 (PMC6007078; doi:10.1186/s12864-018-4772-0)
Supplement: Supplementary file 1 — Supplemental methods for the analysis of the olfactory epithelium data and supplemental figures 1-20. (ZIP 34910 kb) [file 12864_2018_4772_MOESM1_ESM.zip › FIGURE-S5.pdf]

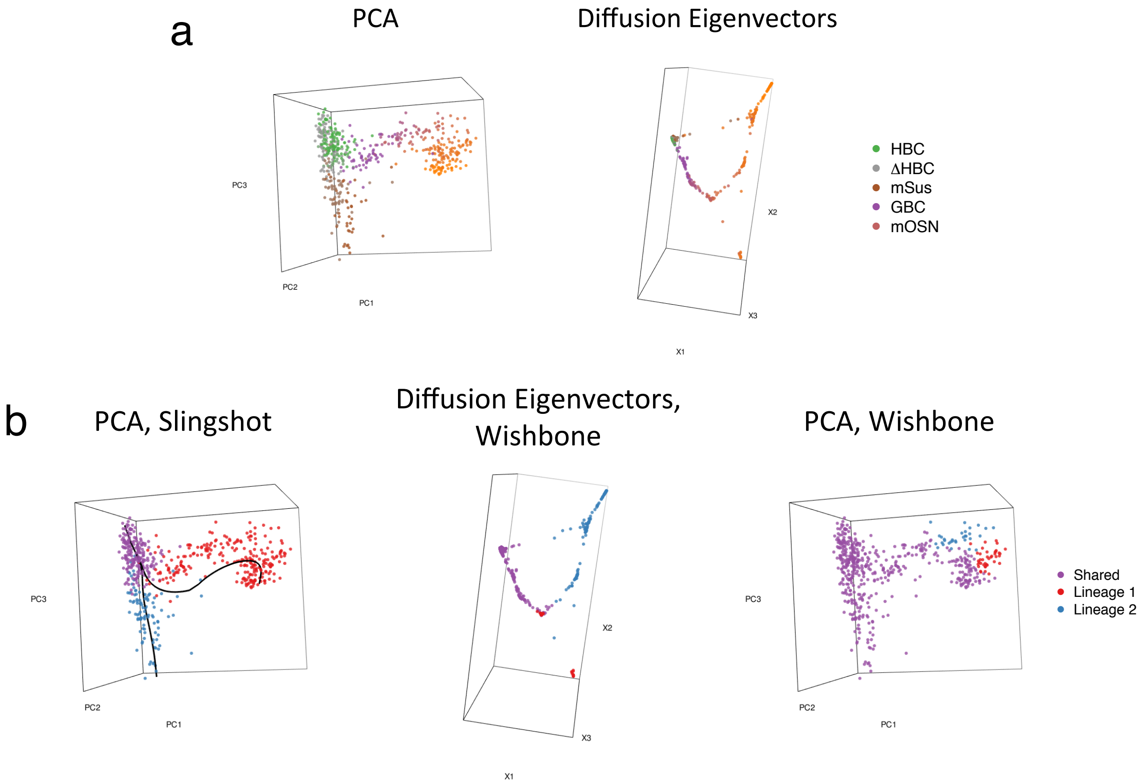
a
PCA
Diffusion Eigenvectors
PC3
PC2
PC1
X2
X3
X1
HBC
ΔHBC
mSus
GBC
mOSN
b
PCA, Slingshot
Diffusion Eigenvectors, Wishbone
PCA, Wishbone
PC3
PC2
PC1
X2
X3
X1
PC3
PC2
PC1
Shared
Lineage 1
Lineage 2
