# Supplementary material for: Slingshot: cell lineage and pseudotime inference for single-cell transcriptomics
Source: BMC Genomics. 2018 Jun 19;19:477. doi: 10.1186/s12864-018-4772-0 (PMC6007078; doi:10.1186/s12864-018-4772-0)
Supplement: Supplementary file 1 — Supplemental methods for the analysis of the olfactory epithelium data and supplemental figures 1-20. (ZIP 34910 kb) [file 12864_2018_4772_MOESM1_ESM.zip › FIGURE-S20.pdf]

a

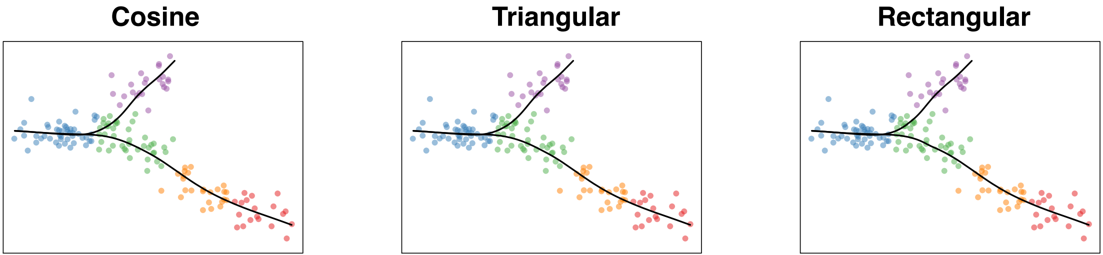

b

	gaussian	epanechnikov	rectangular	triangular	biweight	cosine	optcosine
**gaussian**	1	0.99999469	0.999970993	0.999998398	0.999998043	0.999998682	0.999995799
**epanechnikov**	0.99999469	1	0.99998545	0.999998247	0.999998798	0.999998099	0.999999905
**rectangular**	0.999970993	0.99998545	1	0.999977442	0.999979723	0.999977512	0.999983719
**triangular**	0.999998398	0.999998247	0.999977442	1	0.999999866	0.999999875	0.99999886
**biweight**	0.999998043	0.999998798	0.999979723	0.999999866	1	0.999999873	0.999999286
**cosine**	0.999998682	0.999998099	0.999977512	0.999999875	0.999999873	1	0.999998725
**optcosine**	0.999995799	0.999999905	0.999983719	0.99999886	0.999999286	0.999998725	1
