# Supplementary material for: Slingshot: cell lineage and pseudotime inference for single-cell transcriptomics
Source: BMC Genomics. 2018 Jun 19;19:477. doi: 10.1186/s12864-018-4772-0 (PMC6007078; doi:10.1186/s12864-018-4772-0)
Supplement: Supplementary file 1 — Supplemental methods for the analysis of the olfactory epithelium data and supplemental figures 1-20. (ZIP 34910 kb) [file 12864_2018_4772_MOESM1_ESM.zip › FIGURE-S19.pdf]

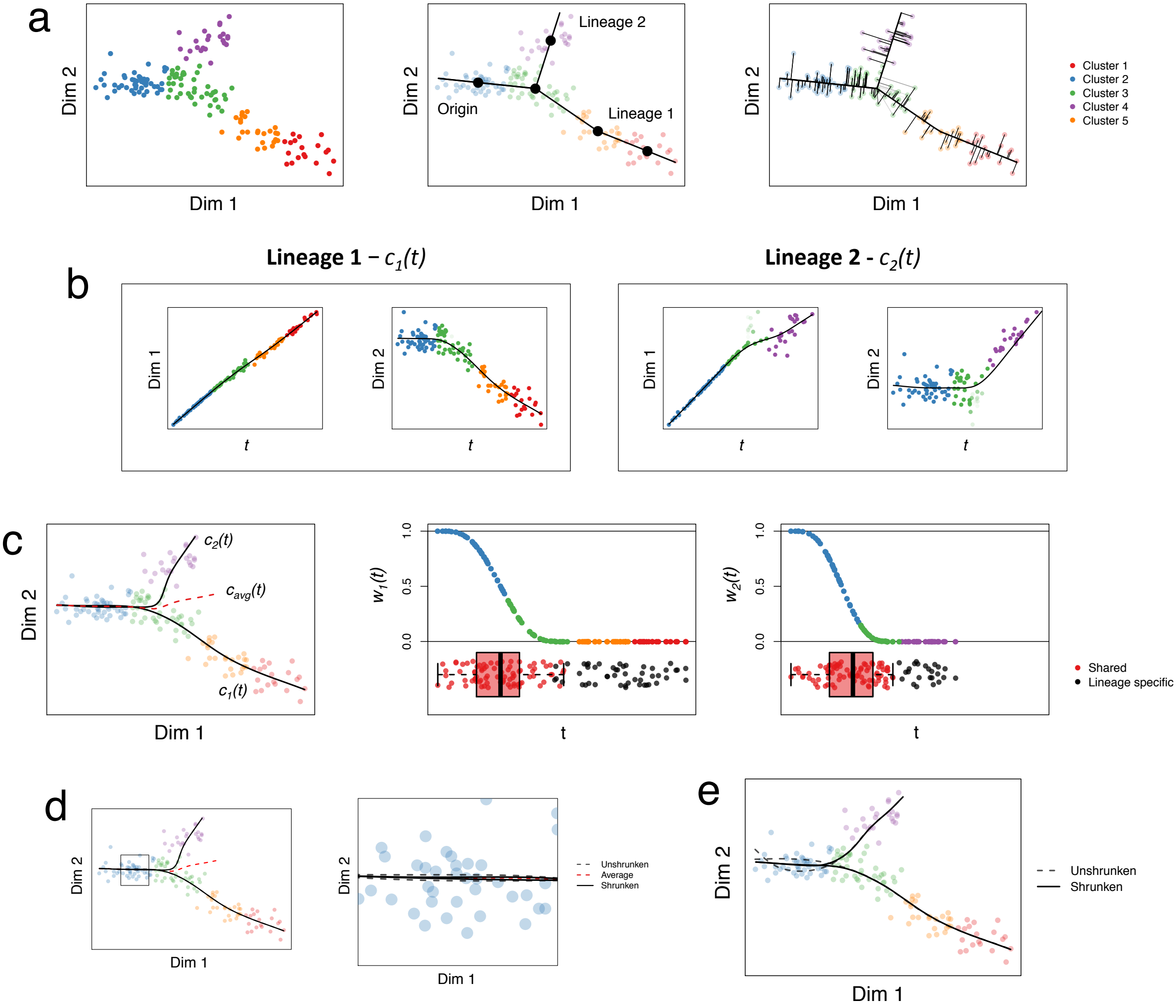
a
Dim 1
Dim 2
Lineage 2
Origin
Lineage 1
Cluster 1
Cluster 2
Cluster 3
Cluster 4
Cluster 5
Lineage 1 – $c_1(t)$
Lineage 2 - $c_2(t)$
b
t
c
$c_2(t)$
$c_{avg}(t)$
$c_1(t)$
$w_1(t)$
$w_2(t)$
1.0
0.5
0.0
Shared
Lineage specific
d
Unshrunken
Average
Shrunken
e
