# Supplementary material for: Slingshot: cell lineage and pseudotime inference for single-cell transcriptomics
Source: BMC Genomics. 2018 Jun 19;19:477. doi: 10.1186/s12864-018-4772-0 (PMC6007078; doi:10.1186/s12864-018-4772-0)
Supplement: Supplementary file 1 — Supplemental methods for the analysis of the olfactory epithelium data and supplemental figures 1-20. (ZIP 34910 kb) [file 12864_2018_4772_MOESM1_ESM.zip › FIGURE-S2.pdf]

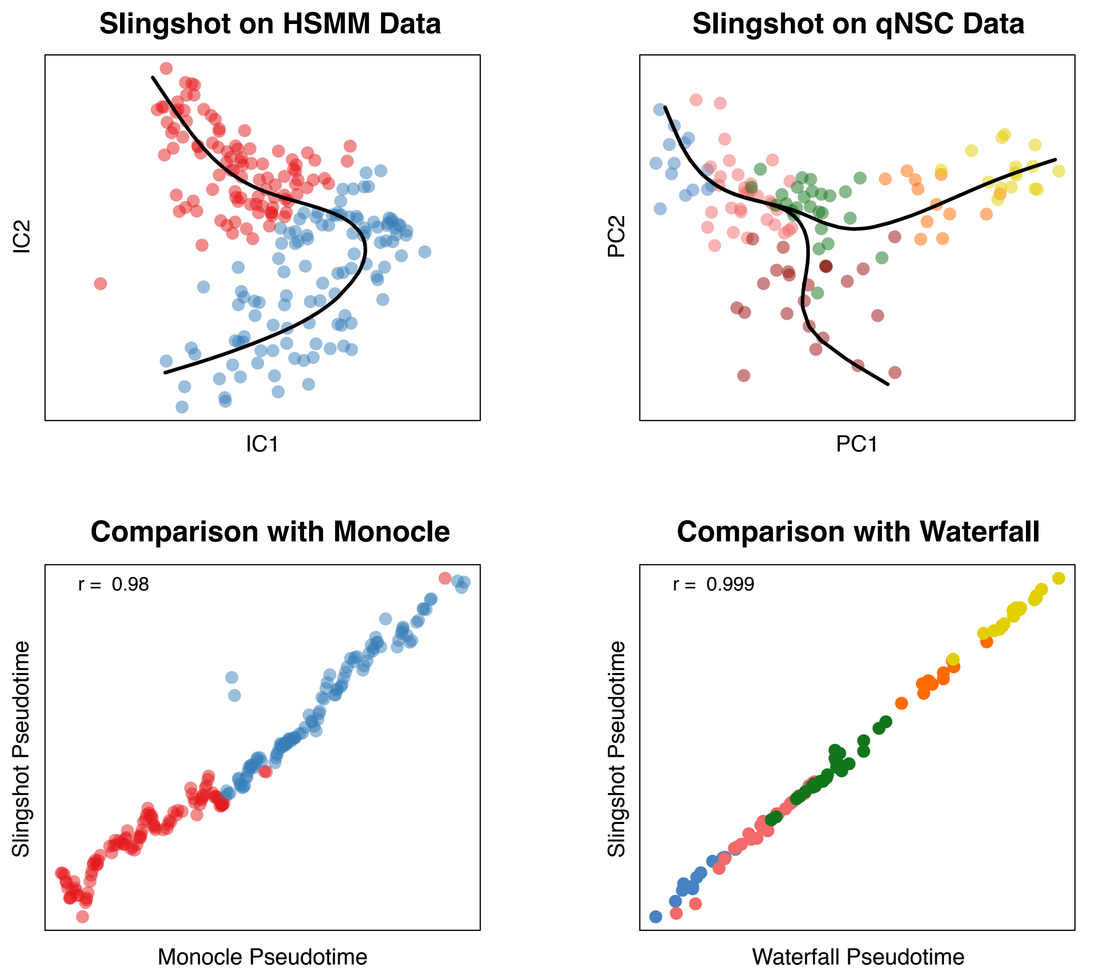

Slingshot on HSMM Data
IC2
IC1
Slingshot on qNSC Data
PC2
PC1
Comparison with Monocle
r = 0.98
Slingshot Pseudotime
Monocle Pseudotime
Comparison with Waterfall
r = 0.999
Slingshot Pseudotime
Waterfall Pseudotime
