# Supplementary material for: Slingshot: cell lineage and pseudotime inference for single-cell transcriptomics
Source: BMC Genomics. 2018 Jun 19;19:477. doi: 10.1186/s12864-018-4772-0 (PMC6007078; doi:10.1186/s12864-018-4772-0)
Supplement: Supplementary file 1 — Supplemental methods for the analysis of the olfactory epithelium data and supplemental figures 1-20. (ZIP 34910 kb) [file 12864_2018_4772_MOESM1_ESM.zip › FIGURE-S16.pdf]

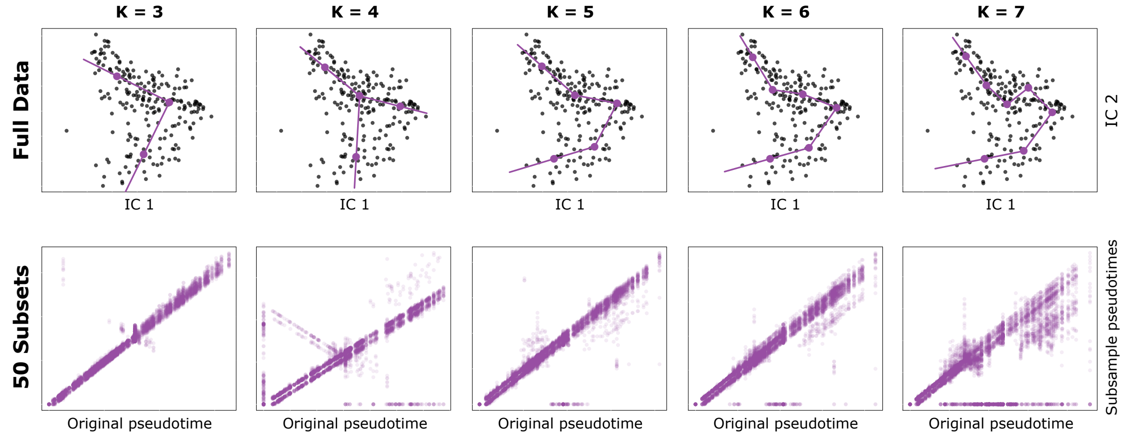
K = 3
K = 4
K = 5
K = 6
K = 7
Full Data
IC 2
IC 1
IC 1
IC 1
IC 1
IC 1
50 Subsets
Subsample pseudotimes
Original pseudotime
Original pseudotime
Original pseudotime
Original pseudotime
Original pseudotime
