# Supplementary material for: Slingshot: cell lineage and pseudotime inference for single-cell transcriptomics
Source: BMC Genomics. 2018 Jun 19;19:477. doi: 10.1186/s12864-018-4772-0 (PMC6007078; doi:10.1186/s12864-018-4772-0)
Supplement: Supplementary file 1 — Supplemental methods for the analysis of the olfactory epithelium data and supplemental figures 1-20. (ZIP 34910 kb) [file 12864_2018_4772_MOESM1_ESM.zip › FIGURE-S11.pdf]

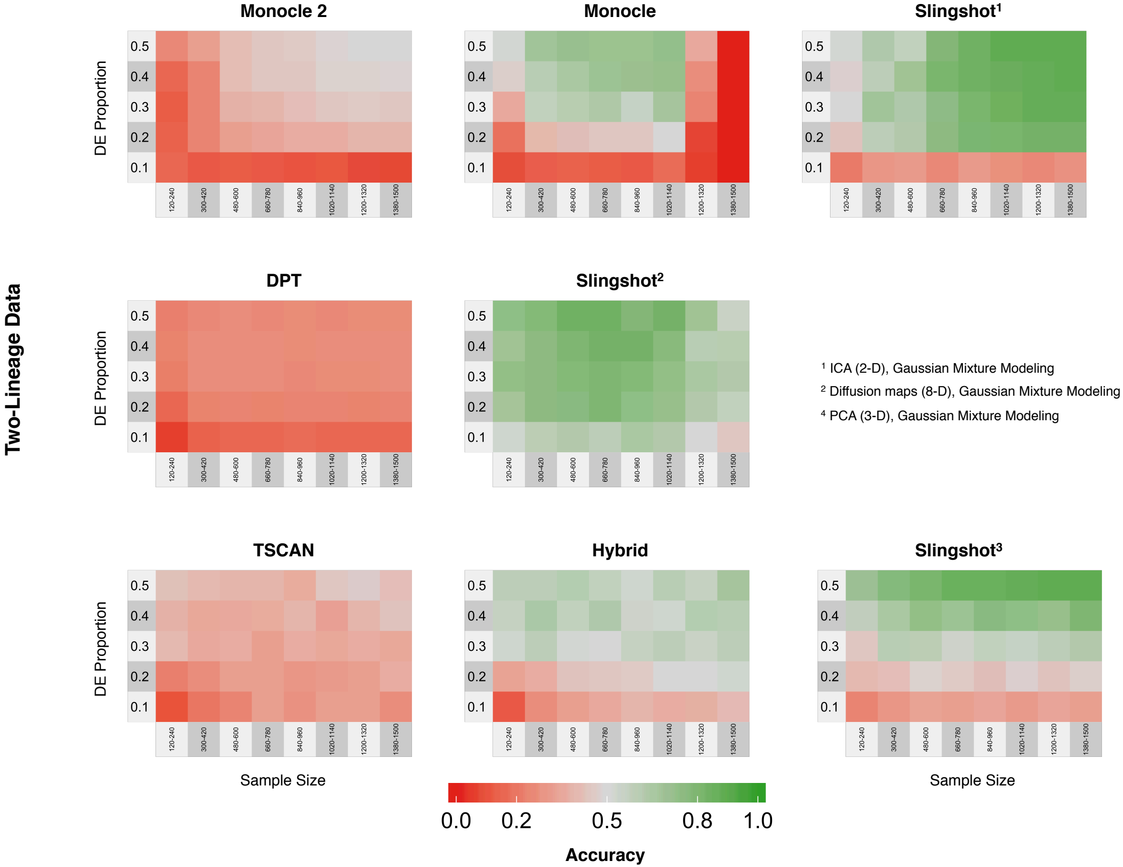

Two-Lineage Data
Monocle 2
Monocle
Slingshot[1]
DPT
Slingshot[2]
TSCAN
Hybrid
Slingshot[3]
DE Proportion
0.5
0.4
0.3
0.2
0.1
120-240
300-420
480-600
660-780
840-960
1020-1140
1200-1320
1380-1500
Sample Size
[1] ICA (2-D), Gaussian Mixture Modeling
[2] Diffusion maps (8-D), Gaussian Mixture Modeling
[4] PCA (3-D), Gaussian Mixture Modeling
0.0
0.2
0.5
0.8
1.0
Accuracy
