# Supplementary figures and images for: Slingshot: cell lineage and pseudotime inference for single-cell transcriptomics
Source: BMC Genomics. 2018 Jun 19;19:477. doi: 10.1186/s12864-018-4772-0 (PMC6007078; doi:10.1186/s12864-018-4772-0)

## PCA

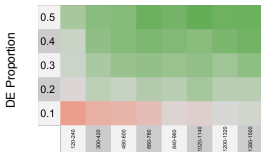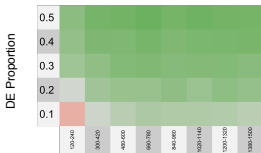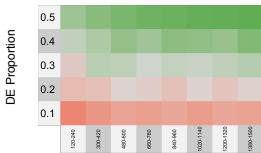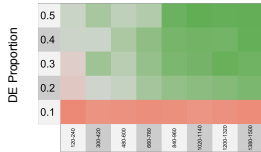

### Sample Size

**ICA**

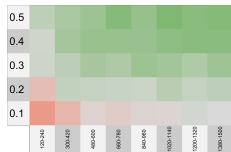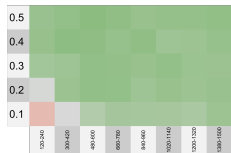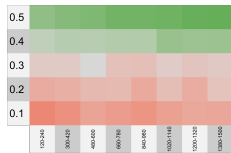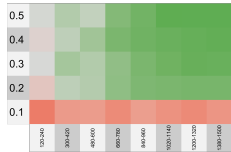

## Diffusion Maps

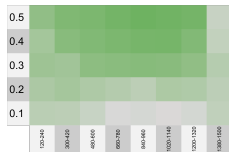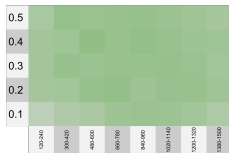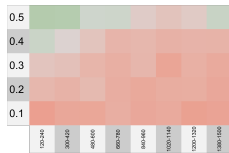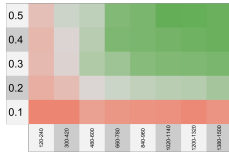

t-SNE

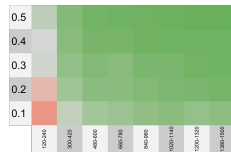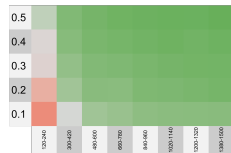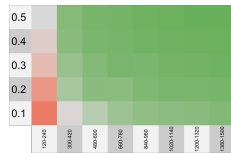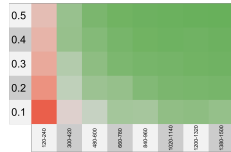

### Sample Size

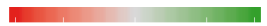

### Accuracy

**5-D**

4-D

### 3-D

## 2-D

Supplement: Supplementary file 1 — Supplemental methods for the analysis of the olfactory epithelium data and supplemental figures 1-20. (ZIP 34910 kb) [file 12864_2018_4772_MOESM1_ESM.zip › FIGURE-S9.pdf]

PCA

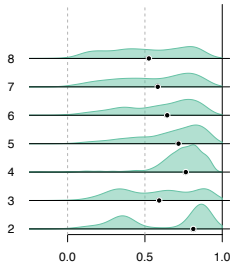

Accuracy

ICA

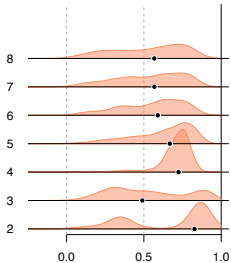

Accuracy

Diffusion Maps

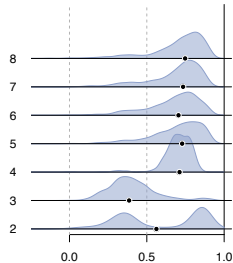

Accuracy

t-SNE

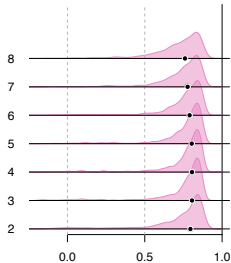

Accuracy

● Median

Supplement: Supplementary file 1 — Supplemental methods for the analysis of the olfactory epithelium data and supplemental figures 1-20. (ZIP 34910 kb) [file 12864_2018_4772_MOESM1_ESM.zip › FIGURE-S8.pdf]

a

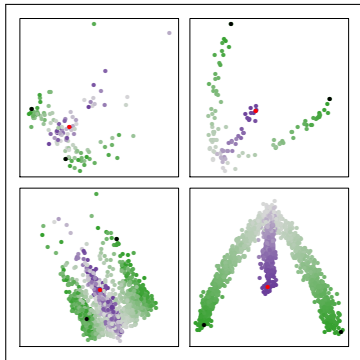

b

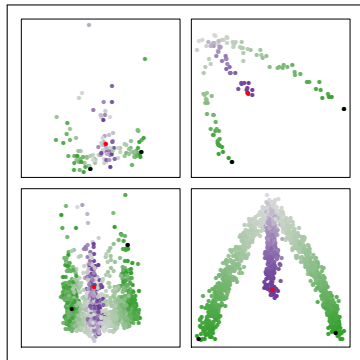

c

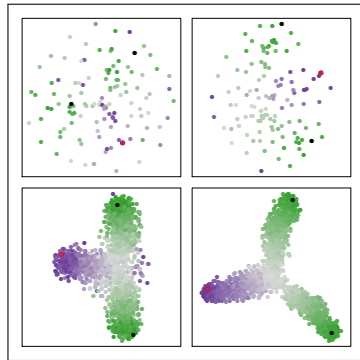

d

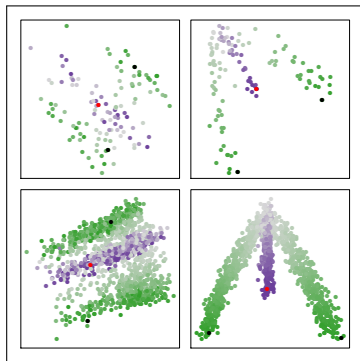

e

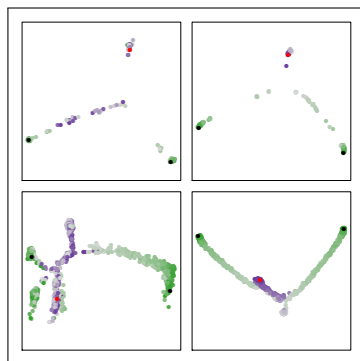

Supplement: Supplementary file 1 — Supplemental methods for the analysis of the olfactory epithelium data and supplemental figures 1-20. (ZIP 34910 kb) [file 12864_2018_4772_MOESM1_ESM.zip › FIGURE-S7.pdf]

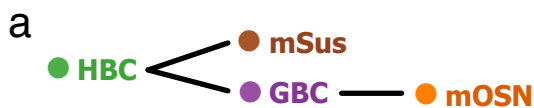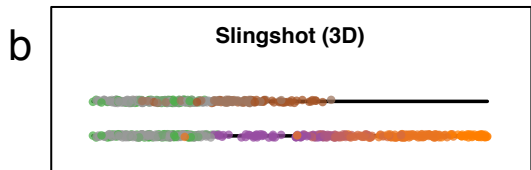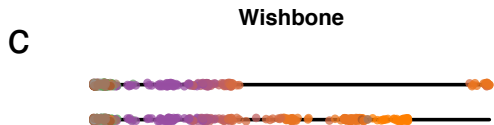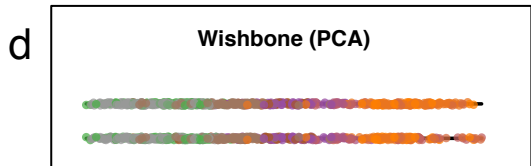

Supplement: Supplementary file 1 — Supplemental methods for the analysis of the olfactory epithelium data and supplemental figures 1-20. (ZIP 34910 kb) [file 12864_2018_4772_MOESM1_ESM.zip › FIGURE-S4.pdf]

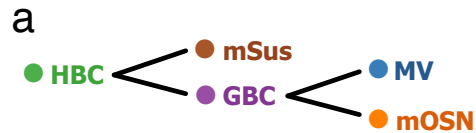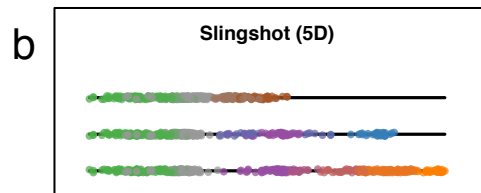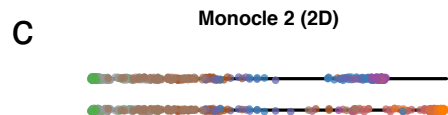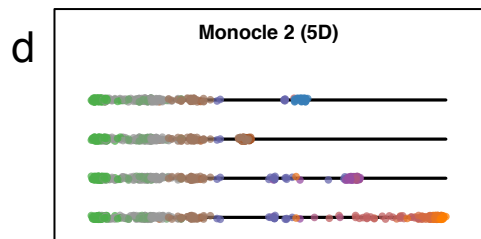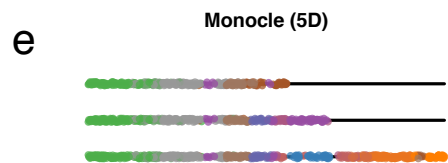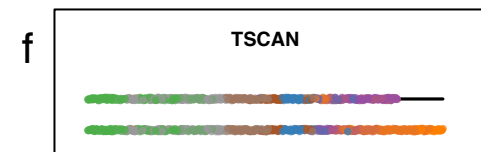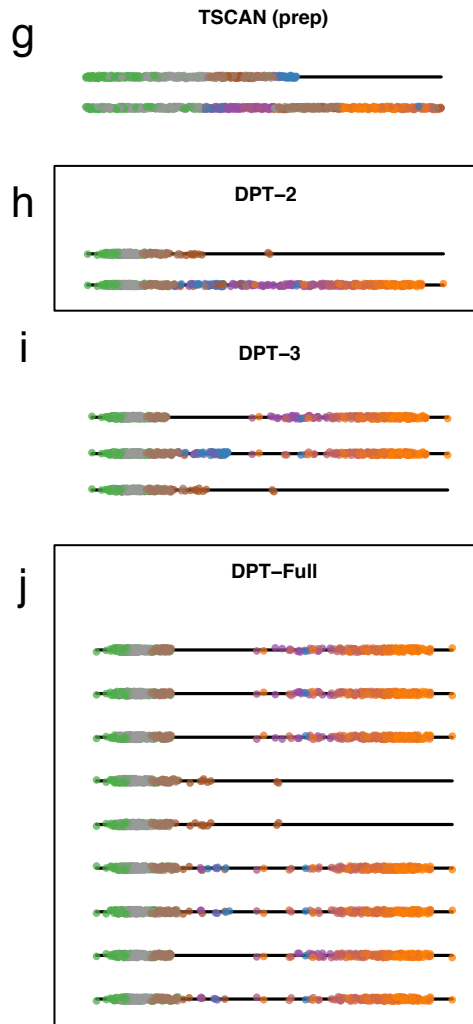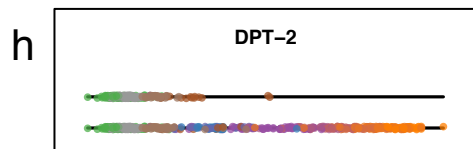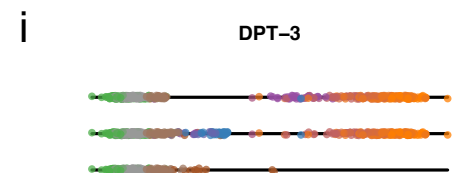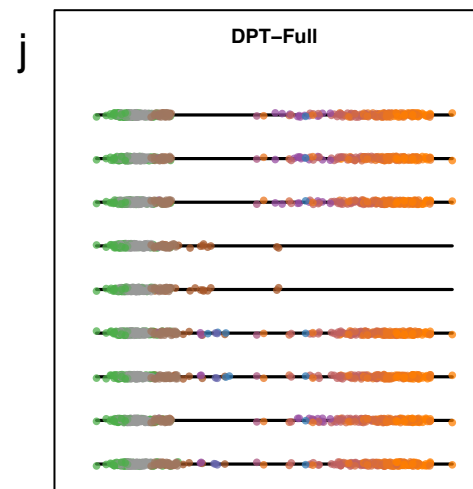

Supplement: Supplementary file 1 — Supplemental methods for the analysis of the olfactory epithelium data and supplemental figures 1-20. (ZIP 34910 kb) [file 12864_2018_4772_MOESM1_ESM.zip › FIGURE-S3.pdf]

### Two-Lineage Data

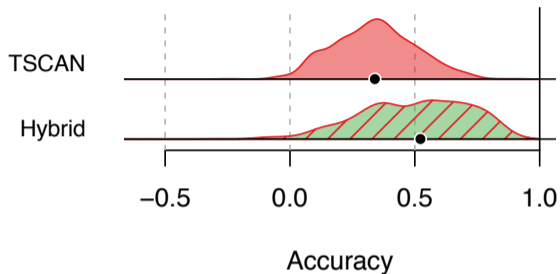

### Five-Lineage Data

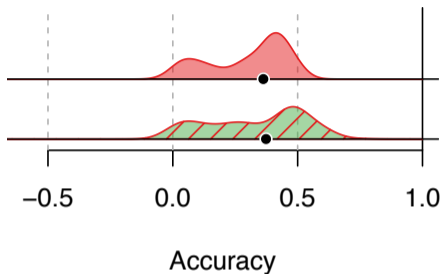

### Difference

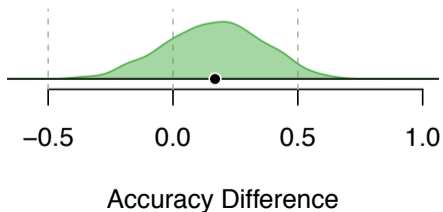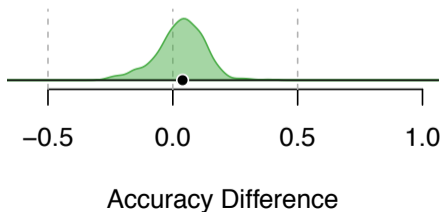

Supplement: Supplementary file 1 — Supplemental methods for the analysis of the olfactory epithelium data and supplemental figures 1-20. (ZIP 34910 kb) [file 12864_2018_4772_MOESM1_ESM.zip › FIGURE-S18.pdf]

a

MST on Clusters

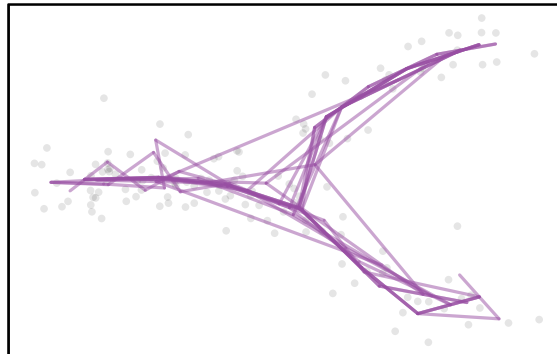 $k = 3-14$ 

MST on Clusters + Simultaneous Principal Curves

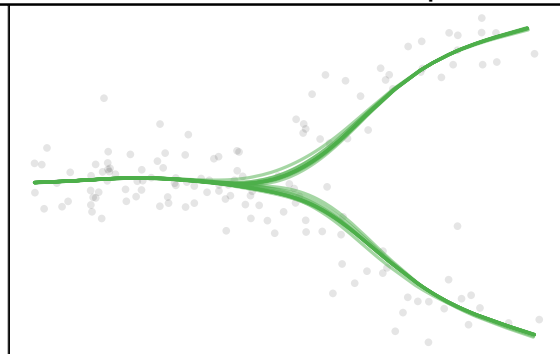 $k = 3-14$ 

b

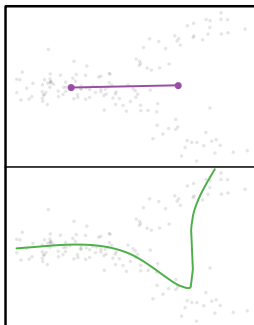 $k = 2$ 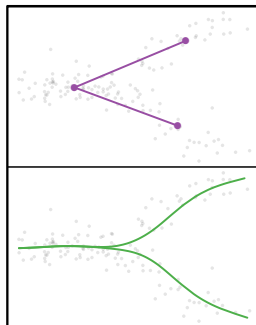 $k = 3$ 

...

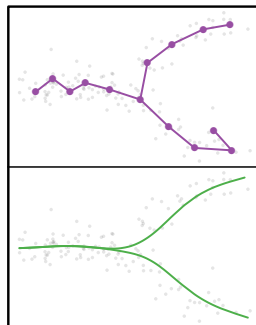 $k = 14$ 

...

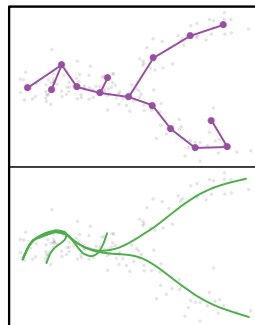 $k = 15$

Supplement: Supplementary file 1 — Supplemental methods for the analysis of the olfactory epithelium data and supplemental figures 1-20. (ZIP 34910 kb) [file 12864_2018_4772_MOESM1_ESM.zip › FIGURE-S17.pdf]

**Monocle 2**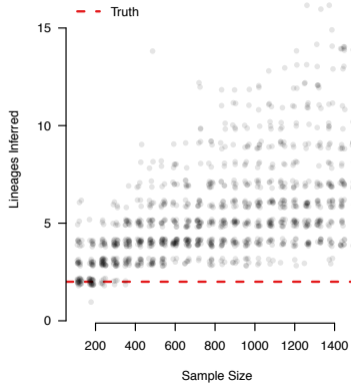**TSCAN**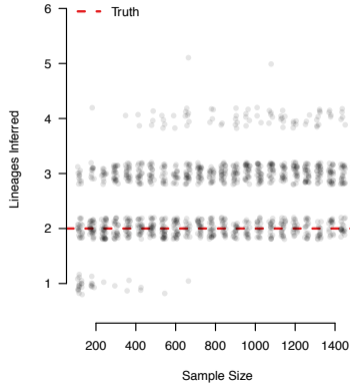**Slingshot**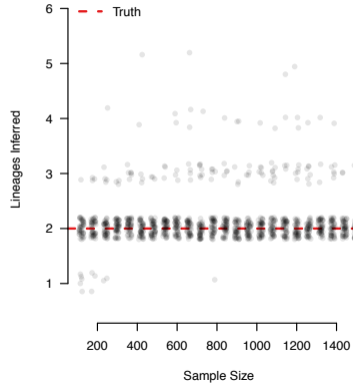

Supplement: Supplementary file 1 — Supplemental methods for the analysis of the olfactory epithelium data and supplemental figures 1-20. (ZIP 34910 kb) [file 12864_2018_4772_MOESM1_ESM.zip › FIGURE-S14.pdf]

# Missing Branch Identities in DPT

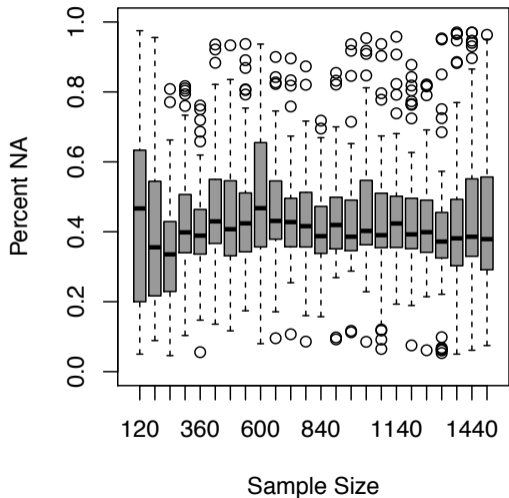

# Missing Branch Identities in DPT

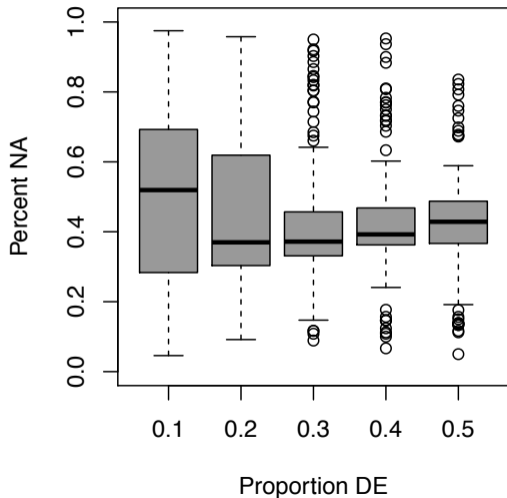

Supplement: Supplementary file 1 — Supplemental methods for the analysis of the olfactory epithelium data and supplemental figures 1-20. (ZIP 34910 kb) [file 12864_2018_4772_MOESM1_ESM.zip › FIGURE-S13.pdf]

# Five-Lineage Data

## Monocle

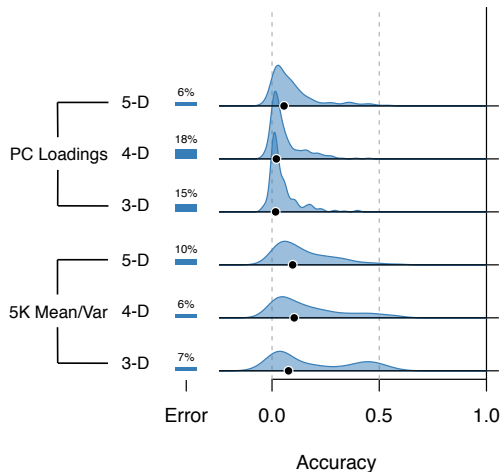

## Monocle 2

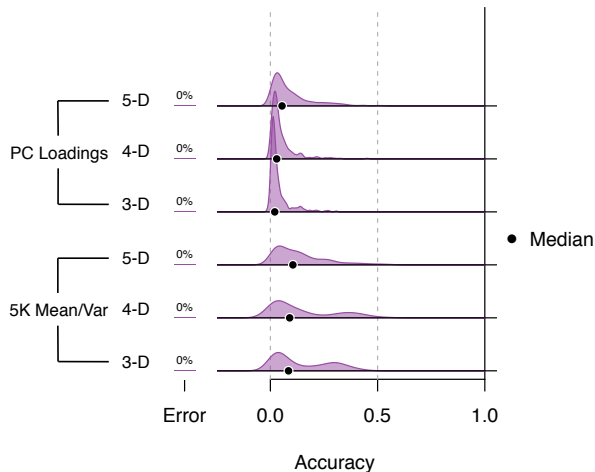

## TSCAN

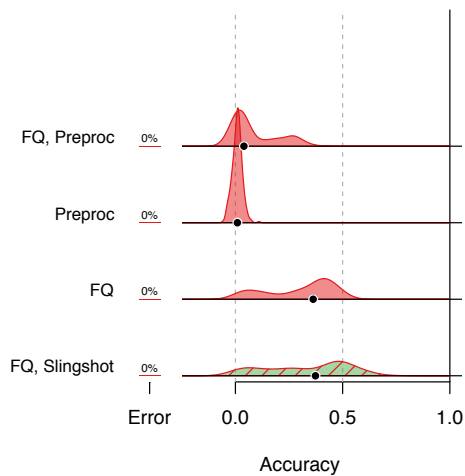

## DPT

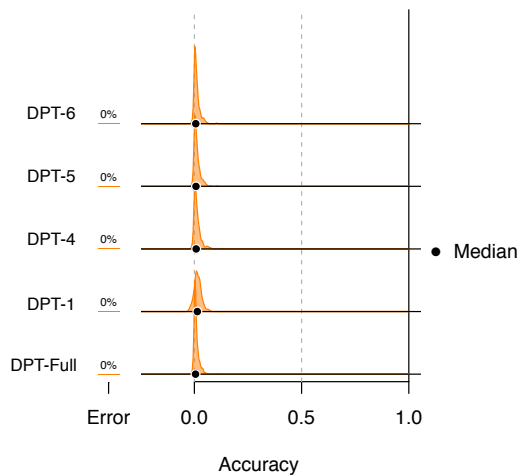

Supplement: Supplementary file 1 — Supplemental methods for the analysis of the olfactory epithelium data and supplemental figures 1-20. (ZIP 34910 kb) [file 12864_2018_4772_MOESM1_ESM.zip › FIGURE-S12.pdf]

# Two-Lineage Data

## Monocle

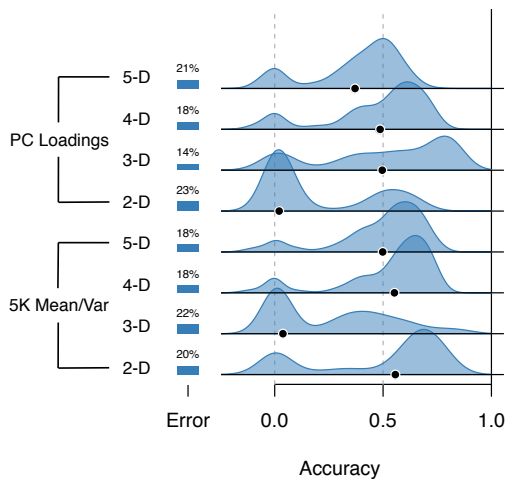

## Monocle 2

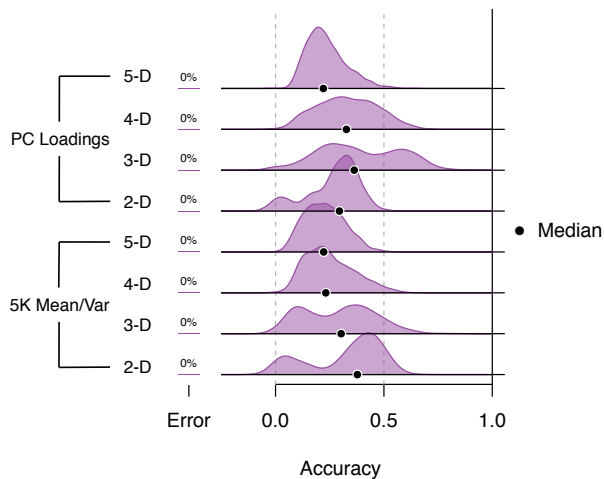

## TSCAN

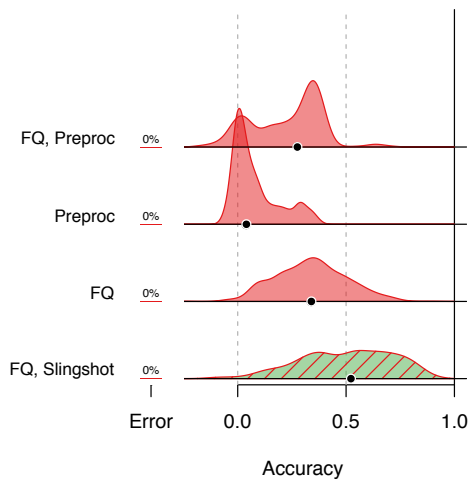

## DPT

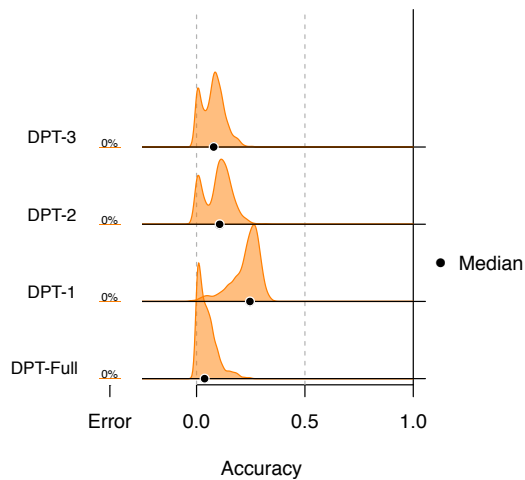

Supplement: Supplementary file 1 — Supplemental methods for the analysis of the olfactory epithelium data and supplemental figures 1-20. (ZIP 34910 kb) [file 12864_2018_4772_MOESM1_ESM.zip › FIGURE-S10.pdf]

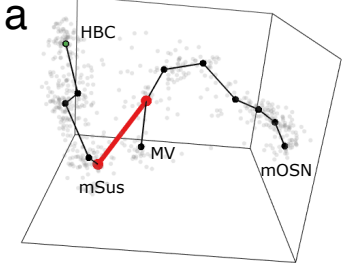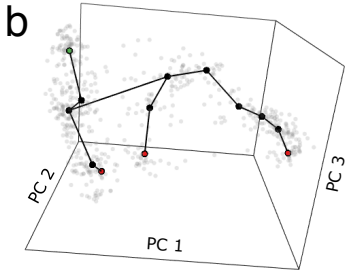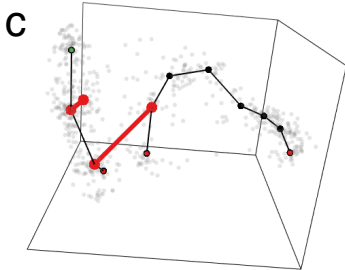

Supplement: Supplementary file 1 — Supplemental methods for the analysis of the olfactory epithelium data and supplemental figures 1-20. (ZIP 34910 kb) [file 12864_2018_4772_MOESM1_ESM.zip › FIGURE-S1.pdf]
